# Ranbow: A fast and accurate method for polyploid haplotype reconstruction

**DOI:** 10.1371/journal.pcbi.1007843

**Published:** 2020-05-29

**Authors:** M-Hossein Moeinzadeh, Jun Yang, Evgeny Muzychenko, Giuseppe Gallone, David Heller, Knut Reinert, Stefan Haas, Martin Vingron

**Affiliations:** 1 Max Planck Institute for Molecular Genetics, Berlin, Germany; 2 Department of Mathematics and Computer Science, Freie Universitat Berlin, Berlin, Germany; 3 Shanghai Chenshan Plant Science Research Center, Chinese Academy of Sciences, Shanghai Chenshan Botanical Garden, Shanghai, China; Centrum Wiskunde & Informatica, NETHERLANDS

## Abstract

Reconstructing haplotypes from sequencing data is one of the major challenges in genetics. Haplotypes play a crucial role in many analyses, including genome-wide association studies and population genetics. Haplotype reconstruction becomes more difficult for higher numbers of homologous chromosomes, as it is often the case for polyploid plants. This complexity is compounded further by higher heterozygosity, which denotes the frequent presence of variants between haplotypes. We have designed ***Ranbow***, a new tool for haplotype reconstruction of polyploid genome from short read sequencing data. ***Ranbow*** integrates all types of small variants in bi- and multi-allelic sites to reconstruct haplotypes. To evaluate ***Ranbow*** and currently available competing methods on real data, we have created and released a real gold standard dataset from sweet potato sequencing data. Our evaluations on real and simulated data clearly show ***Ranbow***’s superior performance in terms of accuracy, haplotype length, memory usage, and running time. Specifically, ***Ranbow*** is one order of magnitude faster than the next best method. The efficiency and accuracy of ***Ranbow*** makes whole genome haplotype reconstruction of complex genome with higher ploidy feasible.

## Author summary

We focus on the problem of reconstructing haplotypes for polyploid genomes. Our approach explored using short read sequence data from a highly heterozygous hexaploid genome. We observed that short read data from strongly heterozygous organisms open up a way for haplotype reconstruction by supplying overlap information between reads. We therefore investigated the role of heterozygosity and ploidy number. Though higher heterozygosity provides more useful reads for reconstructing haplotypes, polyploidy increases the challenge in assembling reads into longer sequences. We called this the problem of “Ambiguity of Merging” fragments. We addressed this problem by designing a new algorithm called ***Ranbow***. ***Ranbow*** was evaluated on real and simulated data from the genomes of tetraploid *Capsella bursa-pastoris* (Shepherd’s Purse) and hexaploid *Ipomoea batatas* (sweet potato). We were able to show that our method achieved high accuracy and long assembled haplotypes in a feasible amount of time, performing at a level consistently superior to other algorithms.

This is a *PLOS Computational Biology* Methods paper.

## Introduction

The rapid advances in sequencing technologies and assembly tools have enabled the assembly of reference genomes from multiple organims [1–3]. Though useful, such reference sequences do not reflect the complex wealth of information in each chromosome entity. Homologous chromosomes are similar, but not identical, and differ in sets of variants. It is therefore clear that these reference sequences are a consensus of homologous chromosomes and are only estimates of them. Single chromosomes are the main components of inheritance. The majority of chromosomal regulatory interactions are derived within chromosomes [4]. Knowing the sequence of single chromosomes provides us with a better view of the genome. The sequence of variants on a single copy of a chromosome is called a *haplotype* [5, 6]. Haplotyping plays an important role in a multitude of biological analysis, such as genome-wide association studies and imputation [7–9], population genetics studies [10, 11], genome regulation [4, 12, 13], and genotype error detection [14, 15].

Current computational methods of haplotype detection for diploid and polyploid genomes use a wide range of data produced by different technologies. ‘Polyploidy’ is a characteristic of genomes with more than two sets of homologous chromosomes. Human, potato, bread wheat, and strawberry have two, four [16], six [17], and eight [18] sets of homologous chromosomes, respectively. One further distinguishes allopolyploidy, where two species hybridize and contribute chromosome sets, and autopolyploidy, where the chromosomes are derived from a single species. In this work we do not make any assumption on the origin of the chromosomes.

Approaches to haplotype detection include haplotype inference from SNP array technologies and population data [19, 20], trio-based haplotyping [21], *de novo* assembly based haplotype reconstruction [22–24], and reference based haplotype reconstruction from sequencing data [5, 25–29]. The latter assumes that reference sequence, aligned reads, and called variants are available as input to construct haplotypes. This is the problem formulation we adopt in our work, focusing on read-based single individual haplotype reconstruction of polyploid genomes.

Multiple combinatorial optimization models aim to address the haplotype reconstruction problem [6]. This includes minimum error correction (MEC) [30], minimum fragment removal (MFR) [31], minimum SNP removal (MSR) [31], minimum fragment cut (MFC) [32], and balanced optimal partition (BOP) [33]. The tools within these models search for a solution, *i.e*. a set of haplotypes, with the highest fitness score of the model. The MEC model, for example, minimizes the disagreement between reconstructed haplotypes and their constitutive reads. Because most of these models are proven NP-Hard [32, 34], heuristic algorithms have been proposed [5, 25, 26]. Though existing models target diploid haplotyping, a few of them can be generalized for polyploid contexts [28, 29].

Diploid and polyploid haplotyping differ in problem complexity. Because the haplotypes in a diploid haplotype are complementary, obtaining one haplotype is sufficient [5, 25]. In contrast, all haplotypes in polyploid genomes must be computed in order to obtain the complete picture of all homologous chromosomes [26, 27]. The step from diploid to polyploid inflates the search space dramatically [28]. The presence of a larger number of homologous chromosomes also leads to the quantitative observation of individual variants. This is called dosage information, which describes the frequencies of variants in a polymorphic site [35]. Errors in dosage information provided as input may also influence the haplotype reconstruction step. Existing programs either expect this information as input or need to determine it.

Moreover, cross-fertilizing or clonally propagated polyploid genomes have been shown to have high heterozygosity [14, 35], meaning that a higher number of variants has to be haplotyped. This is complicated further as the increasing ploidy of an organism causes more information to be pushed into the reference sequence, resulting in lower identity between the reference sequence and either of the chromosomes [14]. This introduces higher error rates in the pre-haplotype reconstruction steps of variant calling and mapping.

Despite these challenges, higher heterozygosity helps to connect more variants from sequence fragments of a certain read size in polyploid genomes. The intervals between variant positions will be shorter, in turn leading to a higher chance of observing two or more variants covered by a read. Only reads covering two or more variants are useful for haplotyping. For instance, the sweet potato genome has an average variant interval length of 58bp [14]; for this genome we have observed that paired-end Illumina whole genome sequencing (2 × 100bp) provides ∼49% useful reads with two or more variants. The same sequencing platform captures about 1% useful reads for an average interval length of ∼1000bp in human (NA12878), meaning that the proportion of useful reads is dramatically higher for the polyploid sweet potato. These characteristics motivate the design of computational tools that use short read sequences for haplotype reconstruction of polyploid genomes with high heterozygosity.

A handful of methods address polyploid haplotype reconstruction. ***HapCompass*** [27] constructs a compass graph, which is a specific type of variant graph, from variants and reads and minimizes the conflicts between reads by finding a spanning tree of the graph. ***HapTree*** [26] works under a probabilistic model, searching for a collection of partial solutions according to a relative likelihood function of reads and a set of candidate haplotypes. ***SDhaP*** [29] constructs a read-based graph where the nodes are the reads and the edges are defined by the overlaps between reads. It uses a semi-definite programming approach that aims to find an approximate solution by a greedy search in the space of all possible haplotype combinations. ***H-PoP*** [28] is a read-clustering based method that models haplotype reconstruction as an optimal read partitioning problem, called the Polyploid Balanced Optimal Partition(PBOP) model. Given a *P*-ploid genome, PBOP clusters the reads into *P* groups with maximized fitness. These methods fall short of capturing all characteristics of polyploid genomes and can be improved in terms of accuracy, memory usage, and running time.

This paper introduces ***Ranbow***, our tool for reconstructing polyploid haplotypes from short reads. We have focused on sites that vary by single-base substitution, multi-base substitutions, and small (<50bp) insertions and deletions. We designed ***Ranbow*** to account for the following domain-specific insights: 1) an identical overlap between two aligned reads does not yield that they stem from one haplotype and may therefore not always be assembled into one haplotype segment, 2) the observed high number of multi-allelic variants must be accounted for, 3) different types of errors need to be accounted for, including errors stemming from base-calling, variant-calling, dosage information, and read-mapping. ***Ranbow*** exploits the dense variants within paired-end reads in highly heterozygous regions to reconstruct haplotypes for all homologous chromosomes in order to account for these domain-specific insights. It clusters the reads and assembles them into short haplotypes on the variant level while considering several error correction schemes to detect, correct, or exclude erroneous variants. ***Ranbow*** continues by haplotype extension of the regions via a multi-partite graph representation, followed by detection of overlaps between haplotypes and paired-end connections. Within this framework, we show that ***Ranbow*** efficiently and accurately constructs long haplotypes using real and simulated data.

## Materials and methods

This section explains the ***Ranbow*** design considerations, defines the haplotype reconstruction problem, presents the algorithm, and describes the generated and simulated data we used in our evaluation.

### Method design considerations

**Fig 1:**
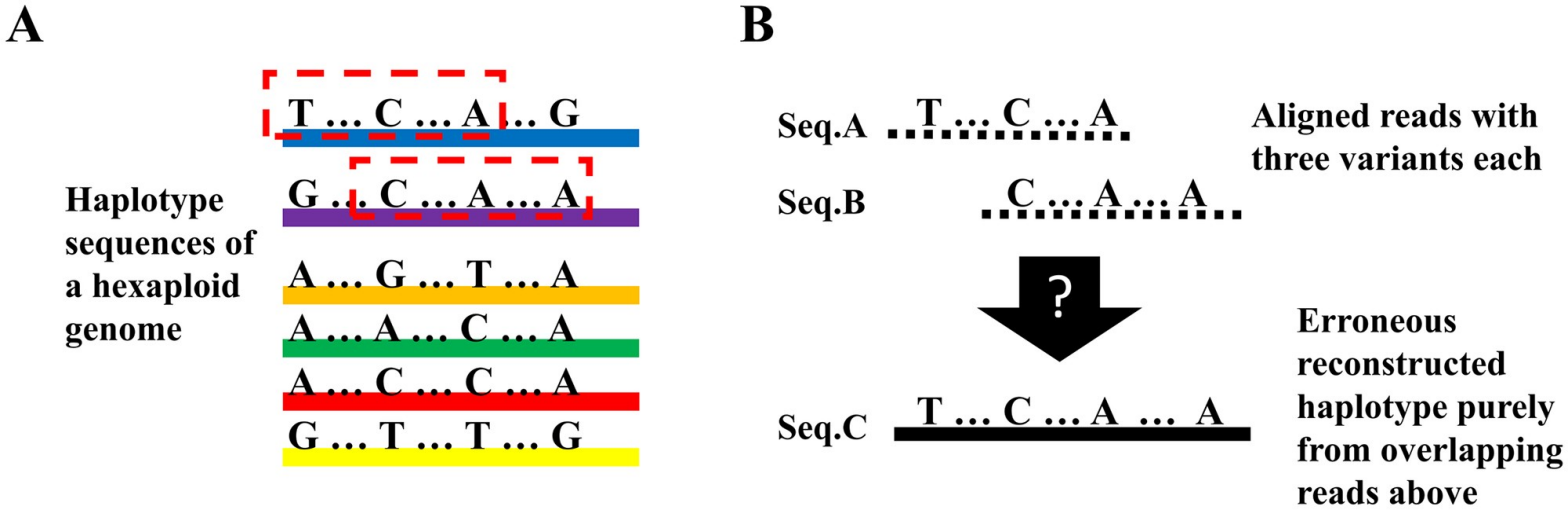
Ambiguity of merging fragments. In a polyploid genome, merging two fragments purely through sequence comparison may result in erroneously reconstructed haplotypes. **(A)** A region of a hexaploid genome with four variant sites. Each color indicates one true haplotype. **(B)** Seq.A and Seq.B are two reads sampled from the selected pieces of the blue and purple haplotypes, respectively. These sequences each carry three variants with an overlap of two variants (*C*…*A*). Merging Seq.A and Seq.B would lead to Seq.C, which, however, is not one of the true haplotypes from **(A)**.

We identified the *Ambiguity of Merging* (*AoM*) fragments problem as one of the major technical challenges specific to polyploid haplotype reconstruction. In diploid genomes, haplotypes complement each other, in the sense that a variant position will allow for one of two possible variants. This ensures that any two overlapping reads with identical variants stem from the same haplotype and therefore the reads can be merged or assembled into one larger haplotype segment. In polyploid genomes the knowledge of the sequence of one haplotype does not suffice to infer the sequence of the other haplotypes. Consequently, any matching overlap between two aligned reads does not yield complete haplotype information (Fig 1) and the two reads cannot automatically be assembled into one haplotype segment. Therefore additional information is needed to determine if two reads stem from the same haplotype.

Our tool deals with multi-allelic variants considering different types of errors. The abundance of multi-allelic variants necessitates integrating these variants in haplotype reconstruction analysis (see *Data* for details). Some available studies exclude multi-allelic variants for the sake of simplicity or reducing the search space. The higher heterozygosity characteristic of a polyploid genome leads to relatively low reference accuracy [14], a challenge for read mapping, variant calling, and dosage estimation [35, 36]. Any error introduced in these steps will transfer to the haplotype reconstruction step. Different types of errors, e.g. base calling and mapping, require separate consideration. Conventional error models consider solely one type of error. For example, the MEC and MFR models consider only variant calling and mapping errors, respectively. It is therefore essential that our new algorithm be able to deal with several types of errors. Some of the available methods, such as ***HapCompass***, require dosage information as input, which is in itself a challenging estimation problem [37].

#### Definitions and problem formulation

Given a *P*-ploid organism, a haplotype defines the sequence of variants in one of the *P* homologous chromosomes. This paper focuses on sites that vary by single-base substitution, multi-base substitutions, and small (<50bp) insertions and deletions; collectively referred to as *Small Polymorphism* (*SmP*). The variants are coded into numbers ranging from zero to *P* − 1, where 0 refers to the reference allele, 1 refers to the first alternative allele, 2 to the second one, and so on. We refer to this as haplotypes in **coded allele space** which is written as: $h_{i}^{\text{Coded}\;\text{allele}\;\text{space}}=h_{i}=a[1].a[2].a[3]\ldots a[N]$, where, *h*_*i*_, *a*[*n*], *N*, and “.” represent the haplotype in coded allele space, the coded variants, the number of polymorphic sites, and the concatenation of the sequences, respectively. Let *F* be the set {*f*_1_, …, *f*_*M*_} of all fragments. A **fragment** *f* is the sequence of variants which are covered by an aligned read. When a read contains a variant not reported in the variant list, the position is considered as a **missing allele** or gap (“−”).

As before [14], we defined the similarity and dissimilarity functions between fragments as:

$$\begin{array}{c} \begin{array}{c} sim\left(f_{1},f_{2}\right)=\sum {}_{n=1}^{N}s(f_{1}\left[n\right],f_{2}\left[n\right])\\ dsm\left(f_{1},f_{2}\right)=\sum {}_{n=1}^{N}d(f_{1}\left[n\right],f_{2}\left[n\right])\;\text{where}\\ s\left(f_{1}\left[n\right],f_{2}\left[n\right]\right)=\{\begin{array}{cc} 1 & f_{1}\left[n\right]=f_{2}\left[n\right]\;\&\;f_{1}\left[n\right]\neq “-”\;\&\;f_{2}\left[n\right]\neq “-”\\ 0 & Otherwise \end{array}\\ d\left(f_{1}\left[n\right],f_{2}\left[n\right]\right)=\{\begin{array}{cc} 1 & f_{1}\left[n\right]\neq f_{2}\left[n\right]\;\&\;f_{1}\left[n\right]\neq “-”\;\&\;f_{2}\left[n\right]\neq “-”\\ 0 & Otherwise \end{array} \end{array} \end{array}$$ (1)

*s*(*f*_1_[*n*], *f*_2_[*n*]) (*d*(*f*_1_[*n*], *f*_2_[*n*])) returns “1” if the two variants *f*_1_[*n*] and *f*_2_[*n*] are available and identical (different). The similarity (dissimilarity) function computes the number of polymorphic sites in which both fragments carry the same (different) variants.

We defined a **haplotype segment** *s* as a consensus obtained from a set of fragments *F*′ computed by the *merge* function as follows:

$$\begin{array}{c} \begin{array}{c} s=merge\left(F^{\prime }\right)=\underset{s^{\prime }}{argmax}\sum_{f_{k}\in F^{\prime }}sim\left(f_{k},s^{\prime }\right)\\ \text{where}\;F^{\prime }\subset F\;\text{and}\;s^{\prime }\;\text{varies}\;\text{over}\;\text{the}\;\text{possible}\;\text{sequences}\;\text{of}\;\text{alleles.} \end{array} \end{array}$$ (2)

We call the elements of the set *F*′ the **supporting fragments** of *s*.

The aim of haplotype reconstruction is to assemble *F* or a subset of *F* into *P* haplotypes *H* = {*h*_1_, *h*_2_, *h*_3_, …, *h*_*P*_}. Note that haplotype reconstruction and haplotype assembly are used interchangeably. In an ideal scenario with availability of sufficient coverage and long error-free fragments, the aim of haplotype reconstruction is to merge fragments into exactly *P* haplotypes. In reality, sequencing errors, mapping errors, and lack of connectivity between alleles mean that haplotypes may only be partially assembled. We define the aim of haplotype reconstruction as reconstructing long haplotypes with high accuracy.

#### Short region haplotype reconstruction

**Fig 2:**
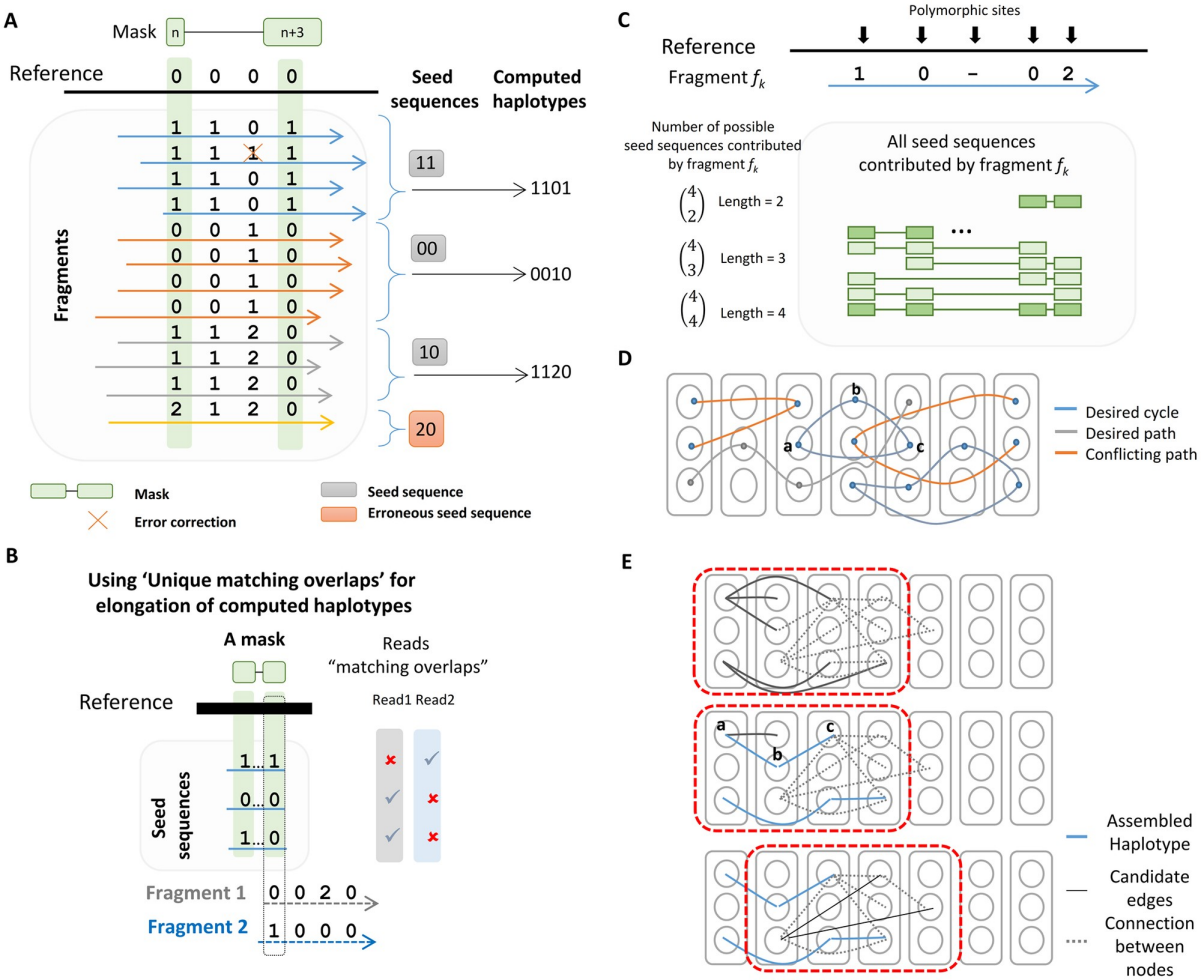
Ranbow’s main processing steps. **(A)** depicts a schematic view of how one mask results in its haplotype segments. The seed sequences cluster the fragments according to the variants they carry. In this figure, there are three clusters; each constructs one haplotype segment. The haplotype segments are supported with four, four, and three fragments, respectively (blue, red, and green fragments). The purple fragment gets discarded because it is detected as an erroneous fragment (see text). **(B)** A unique matching overlap of Fragment 2 and a haplotype block in a triploid genome. Fragment 1 has two matching and one mismatching overlap. This violates the unique match overlap rule. Fragment 2 has one matching and two mismatching overlaps meaning that Fragment 2 can be merged with the first haplotype unambiguously. **(C)** One fragment covering four polymorphic sites contributes eleven seed sequences. The seed sequences of length two, three, and four are shown in the panel in different colors. **(D)** A schematic view of the multi-partite graph and desired and conflicting cycles and paths. **(E)** Schematic view of the steps for constructing haplotypes from desired cycles of length three. A sliding window of length four is depicted in red. Solid gray lines represent the edges starting from the leftmost block of a sliding window. The blue edges are the newly formed haplotypes. The candidate edges at each step are checked to find if they construct a triangle or not. The most supported triangle at each step is converted to one haplotype segment.

A set of aligned fragments differs in their SmP sites. We utilize these differences, or haplotype patterns, to cluster the aligned fragments. In order to cluster the fragments, we have introduced a mask to denote the positions on which to base this clustering. A **mask** (*msk*) is defined as an ordered set of indices of SmP sites, the **seed sequence** of a mask (*t*^*msk*^) is a sequence pattern observed in an aligned fragment at the mask indices. The **supporting fragments of a seed sequence** ($F_{t^{msk_{j}}}$) is a set of fragments which carry identical variants at the mask indices. Fig 2A depicts a schematic view of how we use one mask and its seed sequences to collect haplotype segments of a region. ***Ranbow*** searches for masks which identify a sufficiently large number of unique seed sequences. Each seed sequence clusters together a group of reads which can be merged into one haplotype segment. For instance in Fig 2A, seed sequence “10” groups the green fragments and merges them into the haplotype segment “1120”. *p* unique seed sequences of a mask (e.g, “11”, “00”, “10”) represent *p* unique haplotype segments (“1101”, “0010”, “1120”). This results in a **haplotype block**, or a **block**, of *p* haplotype segments.

#### Haplotype reconstruction on a mask

There are three possibilities for the number of seed sequences (*p*) observed by a mask (*msk*_*j*_) in comparison with the ploidy of the organism (*P*):

***i**) p* = *P*: Because each seed sequence represents one haplotype segment, the fragments containing similar seed sequence stem from one haplotype. We cluster these fragments together. In other words, each cluster can be seen as the supporting fragment of one seed sequence. ***Ranbow*** constructs haplotype segments by merging the supporting fragments of seed sequences.

$$\begin{array}{c} \begin{array}{c} b^{msk_{j}}=\left\{s_{i}^{msk_{j}}|s_{i}^{msk_{j}}=merge\left(F_{t^{msk_{j}}}\right)\right\} \end{array} \end{array}$$ (3)

where $t^{msk_{j}}$ is a seed sequence, $F_{t^{msk_{j}}}$ is the supporting fragments set, $s^{msk_{j}}$ is one of the assembled haplotype segment, $b^{msk_{j}}$ is the obtained haplotype block, and the *merge* function is defined in Eq 2.

The fragments in the supporting fragment set of a seed sequence agree on the variants at the mask sites. Disagreement on the other indices are suggestive of errors that are corrected during the haplotype segment assembly (Fig 2A *red cross*). At the end of this step, a mask with *P* unique seed sequences results in a haplotype block with *P* unique haplotype segments.

***ii**) p* > *P* indicates one or more errors in the supporting fragment of a mask. *P* seed sequences are kept after an error correction step. The erroneous seed sequences are detected according to their fragment support. The smaller the number of supporting fragments, the higher the probability of this being an erroneous seed sequence (Fig 2A *red box*). We deleted the seed sequences deemed erroneous and kept only *P* seed sequences.

***iii**) p* < *P* indicates insufficient information for reconstructing *P* haplotypes of the region. This can result from the similarity of alleles in different homologous chromosomes or from errors from base calling, genome assembly, mapping, and variant calling steps. The algorithm for dealing with these regions is explained in the *Phasing the regions with fewer than P haplotypes* section.

#### Elongating haplotype segments with flanking fragments

To elongate haplotypes that take into account AoM properties, we designed an iterative algorithm utilizing the **unique matching overlaps** of a haplotype in a block, $h_{I}\in b^{msk_{j}}$, and a neighboring overlapping fragment, *f*_*k*_. Fig 2B illustrates a unique matching overlap for a block in a triploid genome. A unique matching overlap has the following properties: ***i***) *f*_*k*_ shares matching overlaps with $h_{i=I}\in b^{msk_{j}}$ meaning *sim*(*h*_*i*=*I*_, *f*_*k*_) > 0 and *dsm*(*h*_*i*=*I*_, *f*_*k*_) = 0, ii) *f*_*k*_ shares mismatching overlaps with $h_{i\neq I}\in b^{msk_{j}}$ meaning *dsm*(*h*_*i*≠*I*_, *f*_*k*_) > 0. In other words *f*_*k*_ shares one matching and *P* − 1 mismatching overlaps with $h_{i}\in b^{msk_{j}}$. This assures that *f*_*k*_ belongs to *h*_*i*=*I*_. At each iteration, ***Ranbow*** selects a fragment and a haplotype with a unique overlap and merges them. The overlapping fragment set is updated by updating the haplotypes. After each iteration, ***Ranbow*** applies the same procedure over the new haplotype list and overlapping fragments set, ultimately resulting in an extended haplotype block.

#### Detecting and evaluating all possible masks

Given *n* polymorphic sites, 2^*n*^ − *n* − 1 possible masks can be computed. It is computationally expensive to find all masks, but not all of the masks are supported by fragments. To obtain all masks with available seed sequences and sufficient fragment support, we designed a two-nested-hash-tables data structure. This data structure helps ***Ranbow*** to pinpoint the masks with fragments supports independent of the size of the scaffolds, distribution of fragments, and the fragment lengths. At the end of this step, ***Ranbow*** provides all masks with available seed sequences and ignores those masks with no supporting fragment.

In the mentioned nested hash tables, the keys in the outer hash table (*OHT*) are mask indices and the values are hash tables. The inner hash table (*IHT*) keeps seed sequences as the key and a list of supporting fragments as the values. S1 Fig Illustrates *IHT* and *OHT* hash tables with an example. To fill this data structure, ***Ranbow*** goes through the fragment list. A fragment with *k* non-missing alleles contributes 2^*k*^ − *k* − 1 seed sequences to different masks. Fig 2C illustrates eleven seed sequences which are contributed with a fragment (*f*_*k*_) consisting of four non-missing alleles. Only masks of length smaller than five are considered in order to decrease the running time. This can be tuned as a parameter.

In order to rank all masks, ***Ranbow*** defines a fitness function which computes the fragment support for the seed sequences of each mask and reports the *P*^*th*^ highest supported seed sequence. In Fig 2A, the seed sequences “11”, “00”, “10”, and “20” are supported by four, three, three, and one fragment, respectively. Three, the number of supporting fragments of “10”, the third highest value (the example genome is triploid, *P* = 3), is reported as the fitness value. ***Ranbow*** ranks the masks according to this fitness function (*MSK*^*ranked*^) and iterates over this ranked list to reconstruct haplotype blocks for these masks. We flag the variants used in reconstructed haplotype blocks as “used”. This includes mask variants and the variants integrated in the elongation step (see *Elongating haplotype segments with flanking fragments*). In the following iteration, we select a mask for which its variants are not flagged “used”.

#### Haplotype elongation via long range connections

Haplotype segments of different haplotype blocks are connected by recruiting fragments that link distant SmPs. These distant connections can be obtained via paired-end, Hi-C, or long read information. We have modeled the problem as a weighted k-partite graph *G*(*V*, *E*, *w*), in which haplotype segments are the nodes and the fragments contributing to two haplotype segments are considered as the edges. In the k-partite graph *G*, *k* refers to the number of constructed haplotype blocks. S2 Fig illustrates haplotype blocks and their corresponding k-partite graph. We define $F^{s_{x}}$ and $F^{s_{y}}$ as the supporting fragments of *s*_*x*_ and *s*_*y*_ haplotype segments and choose $w_{\left(s_{x},s_{y}\right)}=|F^{s_{x}}\cap F^{s_{y}}|$ as the weights of the edges in the graph *G* computed by the number of fragments which contribute to both haplotype segments.

In an ideal scenario of error-free input data, an elongated haplotype segment will correspond to a **desired path**, *i.e*. a path with no conflict among its edges in this graph. A **conflicting path** is one linking two nodes of the same partition. Such a path may be due, e.g., to a mapping error. A conflicting path indicates that two haplotype segments of one block are actually one haplotype, violating the definition of haplotype segments in a block and the definition of partitions in a k-partite graph. We extend the definition of desired path to **desired cycle** when two paths connect two nodes from different haplotype blocks. Fig 2D depicts a schematic view of graph *G*. Every two nodes in a desired cycle are supported by two paths. For instance, for a cycle with length three of *a* → *b* → *c* → *a* (Fig 2D *(Blue)*) every path between a number of nodes, *e.g. a* → *b* → *c*, is supported by another path, *e.g*., *a* → *c*. The elongation step is therefore based on these cycles.

Constructing the whole graph is computationally expensive. First, one needs to find all non-conflicting cycles in the graph. Second, all pairs of segments need to be checked to see if they share fragments in order to add edges. Moreover, the probability of having an edge between distant haplotype segments is low because the read insert size is limited. We have therefore designed a greedy algorithm which avoids constructing the whole graph. ***Ranbow*** sorts haplotype blocks based on their starting positions(*B*^*sorted*^). The starting position of a block is the smallest starting position of its segments. It then defines a sliding window with a user-defined length (ten as default) covering a number of neighboring blocks in *B*^*sorted*^. Fig 2E depicts a sliding window of length four (red rectangles). All edges in this sliding window are constructed and their weights calculated.

For constructing the partial graph in one sliding window, ***Ranbow*** searches for the cycles with length of three, called **triangles**, starting from the first block of the selected sliding window. ***Ranbow*** checks for a pair of edges such that they start from one node, *a*, and end in two nodes, *b* and *c* where *b* and *c* are not in the same block as *a*. ***Ranbow*** then checks whether there is an edge between *b* and *c*. If this edge exists, *a*, *b*, and *c* form a triangle. ***Ranbow*** finds a triangle with **maximum fragment support**, meaning *w*(*a*, *b*) + *w*(*a*, *c*) + *w*(*b*, *c*) is maximum. Having found a triangle with maximum support, ***Ranbow*** merges *a*, *b*, and *c* (Fig 2E *(blue lines)*) and constructs a new haplotype segment by updating *a* and deleting *b* and *c*.

The next step is elongation through direct connections. ***Ranbow*** sorts all edges based on their decreasing weight and iterates over the sorted list. At each iteration, it selects one edge and checks whether merging the two nodes of the edge results in a conflict. In case of no conflict, the nodes are merged into one node and the graph gets updated. The algorithm stops when there are no more edges in the graph, or *E* = ∅. One can assign a predefined threshold on the weights in order to use only highly confident edges, enabling all edges supported by lower than the user-defined threshold to be filtered out. ***Ranbow***’s default threshold is set to three.

#### Phasing the regions with fewer than *P* unique haplotypes

In a *P*-ploid genome, there is the possibility of having regions with *p* < *P* unique haplotype segments. In this situation, *P* − *p* of the haplotypes are identical to one of the other haplotypes. For instance, there are two possible scenarios for a hexaploid genome where *p* = 4 and the unique haplotype segments are {*A*_1_, *A*_2_, *A*_3_, *A*_4_}: either there are two copies of two haplotypes ({*A*_1_, *A*_1_, *A*_2_, *A*_2_, *A*_3_, *A*_4_}) or there are three copies of one haplotype ({*A*_1_, *A*_1_, *A*_1_, *A*_2_, *A*_3_, *A*_4_}). It is essential to be able to distinguish which haplotype has more than one copy. It is also important to note that the unique matching overlap operation (see *Elongating haplotype segments with flanking fragments*) is not applicable to haplotypes with more than one copy. ***Ranbow*** is able to assemble the *p* haplotype segments obtained from one mask into a haplotype block of *p* haplotypes by skipping the elongation with flanking fragments step. ***Ranbow*** sorts the masks based on the number of their seed sequences in descending order, iterating over them to assemble new blocks of haplotypes. This results from ***Ranbow*** finding the largest number that the haplotypes of the region can be reconstructed to. It starts from *p* = *P* − 1 and counts down to *p* = 2. For each *p*, it iterates over sorted masks and checks whether the region is already reconstructed, or partly reconstructed, into its haplotypes. If the haplotypes of a mask region are not yet reconstructed, ***Ranbow*** collects *p* haplotypes to a new haplotype block.

### Data

We used simulated and real datasets to evaluate the competing methods. First we introduce how test datasets are generated.

**SIM data**: To construct a **SIM** dataset we use a genomic scaffold to simulate different ploidy levels with various heterozygosity rates. We employ Haplogenerator [38] to construct different haplotypes with given heterozygosity rate from the scaffold. Haplogenerator [39] uses a Poisson process to randomly generate variant positions. We use ART [40] to simulate short reads with various insert sizes from simulated haplotypes. ART imitates the sequencing process using an empirical error model or quality profiles summarized from large recalibrated sequencing data. We align the simulated reads to the 100kb scaffold followed by variant calling using BWA-MEM [41] and FREEBAYES [42], respectively. The scaffold, aligned reads (BAM format), and called variants (VCF format) serve as input for haplotype reconstruction, while the haplotypes serve as ground truth.

**CBU data**: For tetraploid example we chose Shepherd’s purse, *Capsella bursa-pastoris* (“CBU”). We downloaded the CBU genome and its Illumina reads [43]. We generate BAM and VCF files with BWA-MEM [41] and FREEBAYES [42], respectively, to produce genotype information from the aligned reads. Within a genotype, alleles get randomly assigned to simulate four haplotypes. The subsequent steps are the same as described above (see also S1 Text for more details).

**SP data**: We use the hexaploid sweet potato, *Ipomoea batatas*, genome to produce both simulated and real datasets. The simulated dataset is produced following the same procedure explained for CBU. The real datasets include Illumina short reads of various insert sizes and Roche 454 GS FLX+ pyrosequencer. These raw data were previously published by us [14], and were polished and transformed into standard form, i.e. coded allele space, for haplotype reconstruction analysis. The Illumina data consists of paired-end and mate-pair reads with selected and non-selected insert sizes. We used aligned Illumina reads (mapped via BWA-MEM [41]) for variant calling with FREEBAYES [42], followed by haplotype reconstruction. Roche 454 GS FLX+ pyrosequencer reads are longer than Illumina reads and we employ them to evaluate the reconstructed haplotypes (S3 Fig). All Roche 454 reads were sampled from the same SP individual. We did not utilize these sequencing data in the *de novo* assembly of the SP genome reference to avoid biases in the assembled reference towards these reads. The characteristics of these sequencing libraries are shown in Table 1. Because sequencing errors are more concentrated at homopolymers and at read ends, we were able to polish the Roche 454 reads to produce higher quality ground truth haplotype sequences by trimming low quality base calls(S3 Fig). The thresholds of 99.7% (Phred = 25) for base quality and 99% for mapping quality (Phred = 20) guarantee that the reads will be aligned correctly and that the variants in the reads represent the true haplotypes.

**Table 1:** Sequence library characteristics.

Platform	Sequencing type	Insert size	QC-passed reads	Mapped rate	Monoploid coverage
Hiseq 2500	PE100	350 bp	345M	94.66%	8x
Hiseq 2500	PE100	950 bp	190M	95.07%	4x
Nextseq 500	PE150	550 bp	837M	94.70%	26x
Nextseq 500	PE150	-	694M	91.65%	21x
Hiseq 4000	PE100	20kb	316M	95.67%	6x
GS FLX+	SE	-	3M	98.91%	0.56x

**Fig 3:**
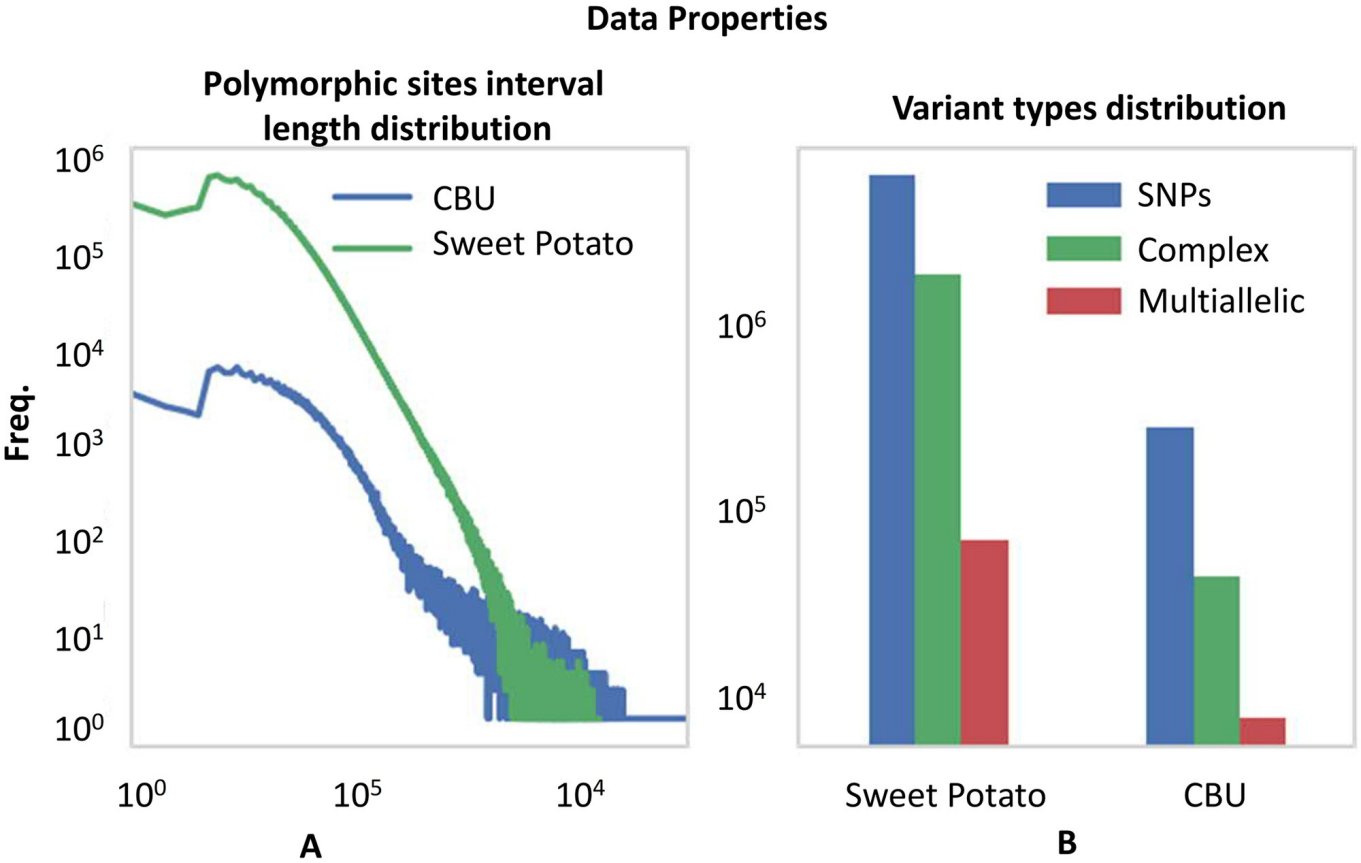
Properties of the sweet potato and CBU genome datasets. **(A)** The interval length distribution of polymorphic sites. Both axes are in log scale. **(B)** The frequencies of different types of variants. The y-axis is in logarithmic scale.

We chose SP and CBU genomes due to the availability of sequence reads sampled from single individuals, allowing for haplotyping of individual genomes. The characteristics of these genomes, i.e. SmP interval length distribution and the rate of variant types, are depicted in Fig 3. We observed very high frequencies of short intervals with exponential drop for larger intervals (Fig 3A). This suggests that the majority of neighboring SmPs can be connected via short read sequencing technologies, such as Illumina. Fig 3B shows the distribution of different types of variants. Though SNP variants are the most frequent type of variants, the number of indels and same-length multinucleotide variants are non-negligible. We therefore allow for all types of variants in our haplotype reconstruction.

**Different size test data**: Some of the available algorithms do not run on large datasets. We therefore defined three datasets distinguished by size for the purpose of testing different aspects of our and competing tools using the procedures explained above (see Table 2).

**Table 2:** Simulated and real datasets for evaluation.

	SIM	CBU	SP	
**Small**	*All*_*a*_	*All*_350*bp*_	*All*_350*bp*_	$All_{b}^{R}$
**Medium**	−	***H-PoP***/***Ranbow***_*c*_	***H-PoP***/***Ranbow***_*c*_	−
**Large**	−	−	−	***Ranbow***^*R*^

**All**: Datasets for evaluation of all competing tools

**R**: Evaluated with Roche 454 reads

**a**: Simulated short reads of 350bp, 500bp, 1kb, and 5kb insert sizes

**b**: Illumina short reads of 350bp, 550bp, 950bp, Non-selected size and 20kb insert sizes

**c**: Simulated short reads of 350bp, 1kb, 2kb, and 5kb insert sizes

*Small datasets*: Those comprise two simulated (*All*_*a*_ and *All*_350*bp*_) and one real ($All_{b}^{R}$) dataset. 1) The *All*_*a*_ dataset is generated according to SIM data scheme, with fixed genome size and insert sizes of 350bp, 500bp, 1kb, and 5kb. We use a 100kb scaffold to simulate datasets with four, six, eight, and ten haplotypes and with 0.001, 0.005, 0.01, 0.05, and 0.1 heterozygosity rates. We produce ten replicates for each ploidy level and heterozygosity rate. For each replicate, we then generate 30x monoploid read coverage of each insert size. 2) *All*_350*bp*_ datasets are generated according to the SP and CPU data generation schemes. This time we fix the library insert size to 350bps and vary the genome length. We selected five scaffold groups of 10kb, 50kb, 100kb, 500kb, and 1000kb lengths, each of which contains ten scaffolds of the corresponding genomes. We selected these scaffolds based on length and coverage such that they represent the majority of scaffolds in the assembly (see S4 Fig). 3) $All_{b}^{R}$ serves as the real dataset which comprises Illumina short reads of 350bp, 550bp, 950bp, 20kbp, and non-selected size insert sizes as input. S5 Fig illustrates the insert size distribution of Illumina libraries. The reconstructed haplotypes were evaluated with Roche 454 reads (see Table 1).

*Medium datasets*: These consist of five paired-end read insert sizes, namely 350bp, 1kbp, 2kbp, 5kbp, and “All” for the SP and CBU genomes, where the “All” library is made by merging the first four datasets. S6 Fig shows the insert length distributions of simulated datasets. Small and medium datasets agree on underlying scaffolds, their variants, and in the procedure used to simulate reads. They differ in terms of monoploid coverage and insert size. The coverage for the 350bp, 1kbp, 2kbp, and 5kbp insert sizes is 40x. The coverage for the “All” dataset is 160x (4×40x).

*Large datasets*: This dataset consists of whole genome Illumina reads of the SP genome as input. For ground truth we use Roche 454 reads (see Table 1).

## Results

We implemented ***Ranbow*** using Python 2.7. ***Ranbow*** is freely available under a GNU licence. Accepted ***Ranbow*** inputs are a reference sequence in FASTA format, a collection of aligned reads in BAM format, and called sequence variants in the VCF format. Dosage information is not required. ***Ranbow*** returns the list of aligned haplotypes in the BAM format. The haplotypes are represented in coded allele space, depicting only the connection between alleles and ignoring the inter-allelic regions (details in *Definition and problem formulation*). We have introduced the “hap” format file for the haplotypes in coded allele space and their specifics. It contains the haplotypes and their read support information. ***Ranbow*** extracts a list of fragments from input sequence reads and variants. The coded allele space format allows the volume of the read-file to be significantly reduced. For example, we obtained 1.6Gb of aligned fragments from 657Gb of mapped reads in sweet potato genome data (see *Data*). Data distribution was automated to multiple cores. ***Ranbow*** accepts the number of available cores as an input parameter, grouping scaffolds to minimize the estimate of maximum running time for the busiest core. This feature is invaluable when dealing with high depth sequence reads and whole genome sequence data applications.

We evaluated ***Ranbow*** (V2.0) against ***HapCompass***, ***SDhaP***, and ***H-PoP***. ***HapTree*** could not be included due to runtime error on some of the datasets. ***Ranbow*** and ***HapCompass*** accept BAM and VCF format as input and produce aligned haplotypes in the BAM format. Pre- or postprocessing the data in order to apply the algorithm is unnecessary. ***H-PoP*** and ***SDhaP*** accept a file format which contains allele connectivity via fragments, and produce a similar format output file. The application of these methods on real data requires pre- and postprocessing steps for the tools to accept the data as input (see S2 Text). ***HapCompass*** and ***Ranbow*** are the only methods that can handle non-SNP sites. ***SDhaP*** cannot handle non-SNP polymorphic sites if the number of alleles is higher than four, a restriction that needs to be considered in the pre-processing step for this method. ***H-PoP*** only integrates one reference and one alternative allele. It converts the rest of alternative alleles to either the reference or the first alternative alleles (see S2 Text).

**Fig 4:**
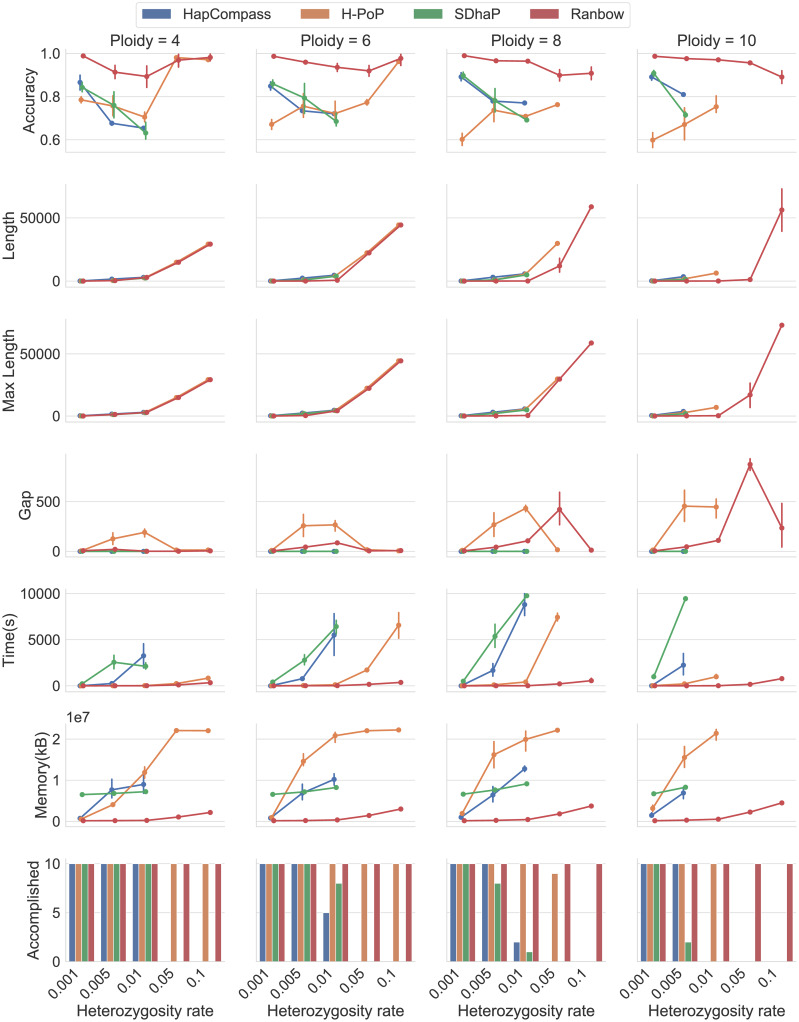
Comparison of methods on *All*_*a*_ datasets. Evaluation of competing methods in terms of accuracy, average haplotype length, maximum haplotype length, gap length (these lengths are in coded allele space), running time, and memory usage on *All*_*a*_ datasets. *All*_*a*_ contains 200 datasets, each with a heterozygosity rate of 0.001, 0.005, 0.01, 0.05, or 0.1 and a ploidy level of four, six, eight, or ten. Columns and rows represent the ploidy level and evaluation metrics, respectively. Each point is the average of up to ten datapoints, each the result of one method on one dataset. For better distinguishability of the methods, lines connect the points for each method. An accuracy (or a length) datapoint represents the average accuracy (length) of all assembled haplotypes in a dataset. A time datapoint (memory or max length) represents the running time (peak memory usage or maximum haplotype length) of a method on a dataset. Bar plots in the last row show the number of replicate data sets for which a method accomplished computation of a result.

Some of the available algorithms did not finish in reasonable time on large datasets. We therefore constructed three groups of datasets with different sizes as described in *Data* above. We started by comparing all methods on **Small** datasets. Since ***H-PoP*** delivered haplotyping results in most cases, we compared it with ***Ranbow*** using **Medium** datasets. Only ***Ranbow*** obtained results on **Large** datasets. As evaluation metrics we use accuracy, haplotype length, running time, and memory usage. The accuracy is the generalized form of reconstruction rate [44] defined for diploid genome. Each reconstructed haplotype is assigned to one and only one ground truth haplotype based on sequence similarity. For these assignments, the accuracy reports the ratio of the number of correctly reconstructed variants over the length of the reconstructed haplotype in coded allele space, where the length does not count missing alleles. Fig 4 shows the evaluation on *All*_*a*_ datasets. *All*_*a*_ contains 20 configurations (four ploidy levels times five heterozygosity rates) and 200 datasets (ten replicates for each configuration). We set the high time limit of 10000 second for this small genomic region. ***SDhaP***, ***H-PoP*** and ***HapCompass*** failed for some configurations mainly when heterozygosity and/or ploidy was high. ***Ranbow***’s accuracy is mostly between 0.9 and 1.0 and it performs best in terms of accuracy. For low heterozygosity (0.001, 0.005, and, 0.01) and ploidy levels, ***H-PoP*** performs better than ***Ranbow*** in terms of haplotype length, although at the cost of lower accuracy. ***Ranbow*** and ***H-PoP*** will allow for missing alleles in the result (see fourth row). The gap length is calculated as the number of missing alleles between first and last defined variant. For example in ‘0 − 1 − − 00’, haplotype and gap lengths are four and three, respectively. ***Ranbow*** can handle every heterozygosity rate and ploidy in a reasonable running time. Maximum running time for ***Ranbow*** is 1114 second for ploidy level 10 and heterozygosity of 0.1. ***Ranbow*** performs best in terms of peak memory usage as well. Table 3 shows the memory usages of competing methods in GB for 0.01 and 0.05 heterozygosity rates.

**Table 3:** Average peak memory usage on *All*_*a*_ dataset with 0.01 and 0.05 heterozygosity. “-” represent the methods failed to accomplish.

Peak memory usage(GB)								
Heterozygosity rate	0.01				0.05			
Ploidy	4	6	8	10	4	6	8	10
**Ranbow**	0.21	0.32	0.41	0.49	1.01	1.35	1.73	2.15
**SDhaP**	6.88	7.84	8.70	-	-	-	-	-
**H-PoP**	11.33	19.86	19.00	20.40	21.05	21.01	21.13	-
**HapCompass**	8.56	9.73	12.18	-	-	-	-	-

In the absence of a ground truth, models such as MEC or MFR (see Introduction) serve as evaluation metrics. For example, the MEC metric computes the MEC scores between the reconstructed haplotypes and their corresponding fragments. ***HapCompass***, ***SDhaP***, and ***H-PoP*** use the MEC metric. The downside of assessing tools with these metrics is that they fail to evenly capture the benefits these tools offer. The MEC metric only considers sequencing errors and the MFR model only the mapping errors.

For this paper we use Roche 454 reads for evaluating the assembled haplotypes in the real datasets. We did not use these reads for haplotype reconstruction. Roche 454 reads are on the order of 800 bp long S3 Fig. For designing an evaluation metric we proceed as follows. A 454 read gets aligned to the reference sequence. Then the haplotype to compare the 454 read to is chosen based on maximal number of matches and minimal number of mismatches between the 454 read and overlapping haplotypes. In particular, a 454 read with a switch error will have many mismatches with respect to the haplotype.

**Fig 5:**
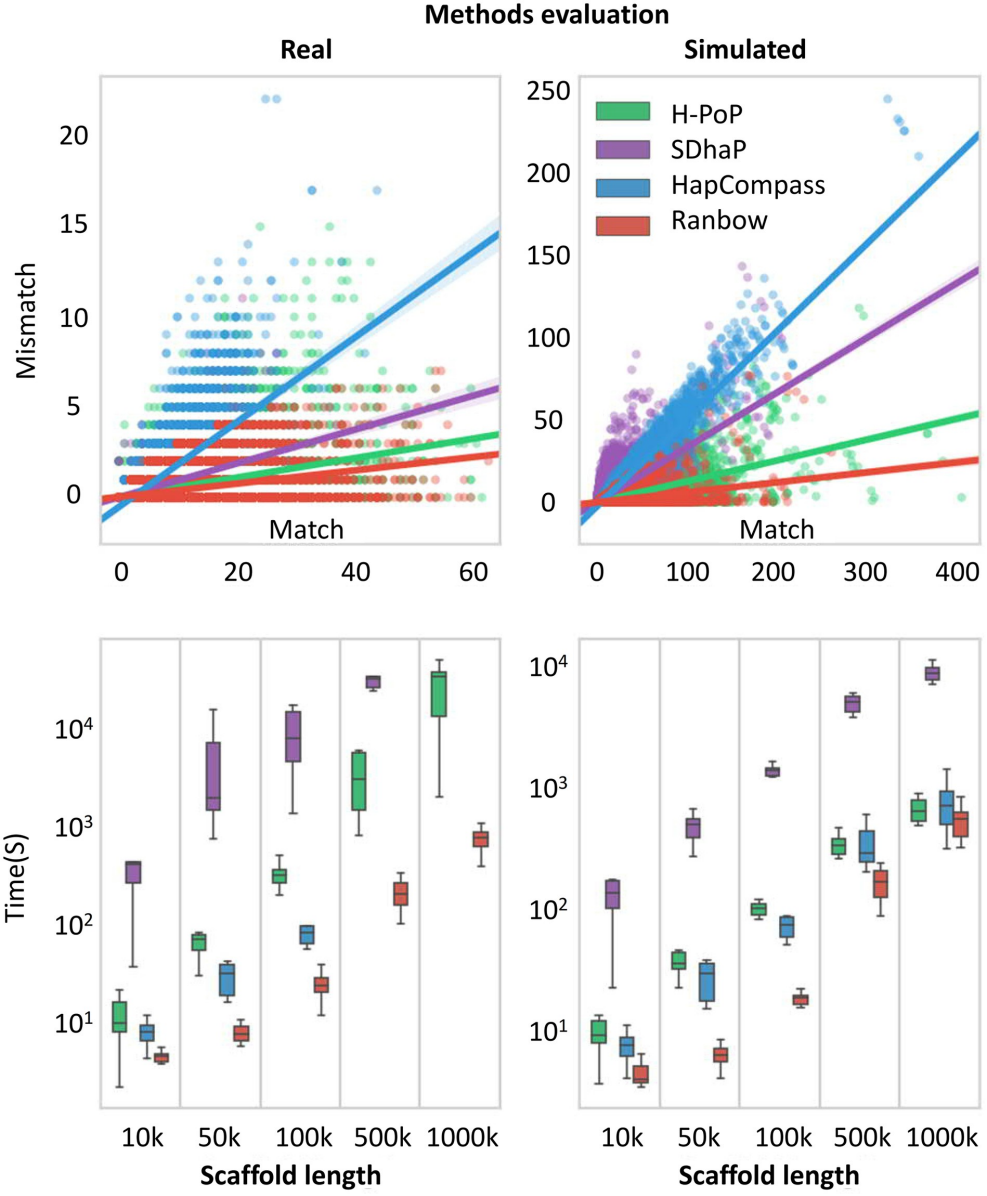
Comparison of all methods on sweet potato real and simulated datasets. A, B) Match-mismatch plots for sweet potato real and simulated datasets, respectively. These scatter plots illustrate the accuracy of the methods. C, D) The execution time s for different scaffold size groups (10 scaffolds per five scaffold length groups, totally 10×5 scaffolds). The y-axis is in logarithmic scale.

We further integrate haplotype accuracy and length through a “match-mismatch plot”, our new approach that evaluates and visualizes the performance of various methods (shown in Fig 5A and 5B). Each dot in a match-mismatch plot corresponds to one assembled haplotype that is compared to a ground truth. The reconstructed haplotype gets assigned to the ground truth and the number of matches and mismatches are calculated. The x-coordinate (y-coordinate) of a dot indicates how many alleles are assembled correctly (incorrectly). Missing alleles are not considered at this point. The observation frequency for each individual haplotype is encoded in the shading at the respective (*x*, *y*) position. Because the aim of haplotyping lies in obtaining longer and more accurate sequences, a larger x-value and smaller y-value are desired. The lines in the plot depict linear fits to the dots generated by each method. The flatter the slope of the line, the higher the overall accuracy of the method. The match-mismatch plot simultaneously evaluates and visualizes accuracy and length of a haplotype.

The results of $All_{R}^{b}$ and *All*_350*bp*_ evaluation are shown in Fig 5. The match-mismatch plot for real data is presented in Fig 5A. x-axis values correspond to the number of variants covered by Roche 454 reads (see S7 Fig). Fig 5B shows the match-mismatch plot for *All*_350*bp*_. We evaluated the accuracy in addition to match-mismatch plot in S8 Fig. Judging by the slope of the regression lines in the match-mismatch plots (Fig 5A and 5B) and the reported accuracy (S8 Fig), ***Ranbow***’s performance is consistently superior with respect to accuracy for real and simulated datasets. S9 Fig evaluates ***Ranbow*** and ***H-PoP*** in terms of haplotype length, this time measured in base pairs. This plot shows the overall equivalent performance of these two methods in terms of haplotype lengths on CBU dataset. Fig 5C and 5D show the execution times for different scaffold size groups. ***Ranbow*** is two (scaffold group 10kb) to 30 (scaffold group 1000kb) times faster than the next best method for real data and also several times faster than the next best method for simulated data. S1 Table shows the running time of competing methods in detail.

**Fig 6:**
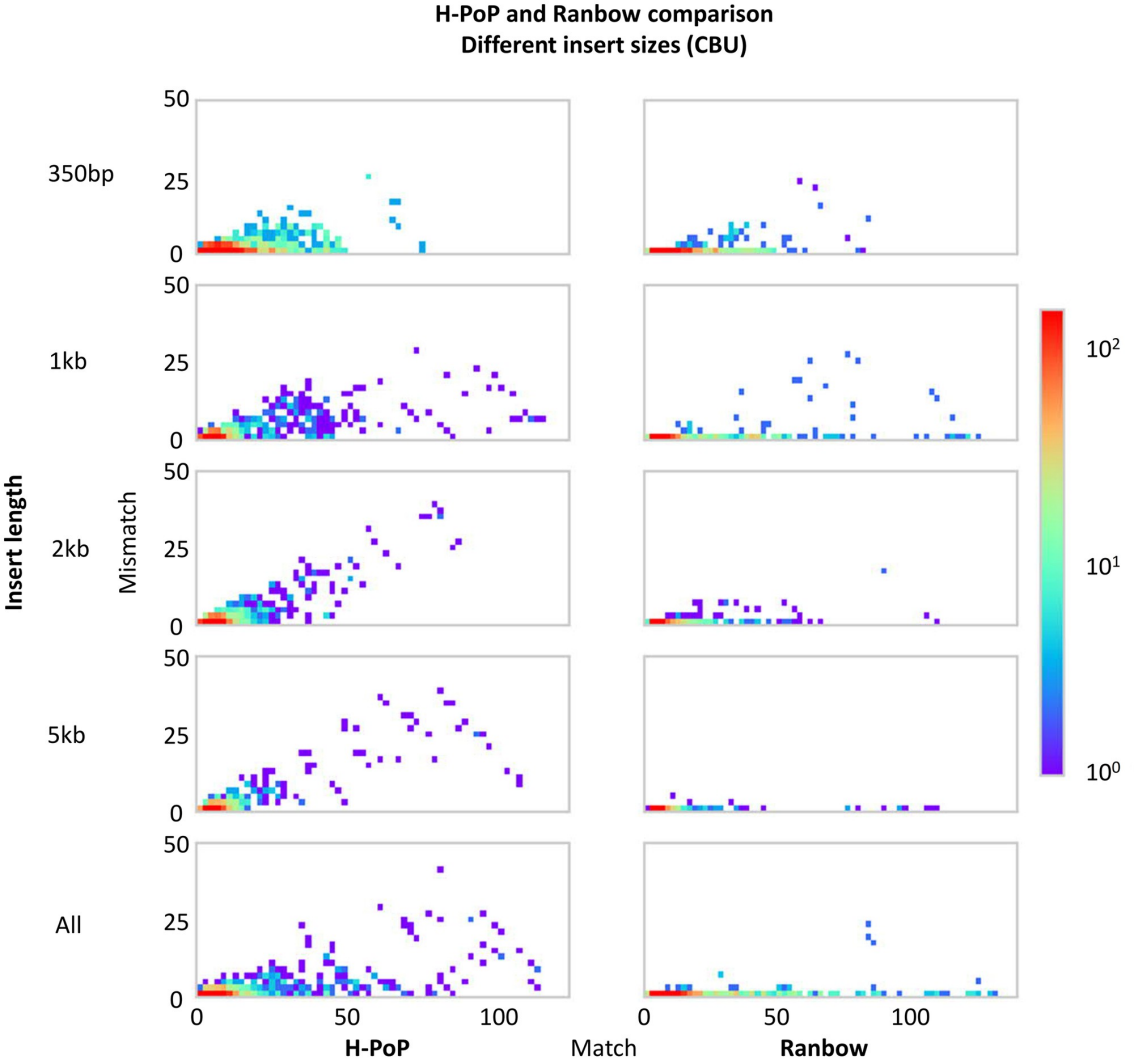
The comparison of *H-PoP* and *Ranbow* on different insert sizes for simulated dataset of CBU genome.

Because some of the datasets did not achieve a reasonable running time on ***SDhap*** and ***HapCompasss***, we focused our medium datasets comparison on ***Ranbow*** and ***H-PoP***. Fig 6 shows the match-mismatch plots for ***H-PoP*** and ***Ranbow*** for different insert sizes on the CBU genome. ***Ranbow*** is more accurate and assembles longer haplotypes. The length and frequency distributions of the variants correctly reconstructed into haplotypes are shown in S10 Fig. Integrating libraries of different insert size is a common scenario when sequencing new organisms. We therefore designed the “All” datasets to test ***Ranbow***’s performance in this situation. Performance increases markedly when different insert sizes are integrated (see Fig 6 *bottom row*). ***Ranbow*** uses the short insert sizes for local haplotype assembly and longer insert sizes for elongation of the haplotypes. Due to the exceedingly long running time on the Large dataset (SP whole genome datasets) by ***H-PoP*** and the other competing methods, we limited our evaluation to ***Ranbow***. S11 Fig evaluates the haplotypes computed from Roche 454 reads. The slope of the fitted line is 0.07 showing ***Ranbow***’s superior performance.

## Discussion

***Ranbow*** outperforms the competing methods for the following three reasons:

1. Integrating multi-allelic variants: ***Ranbow*** has an advantage over methods that search the solution space. Multi-allelic variants substantially inflate the solution space of possible haplotypes. ***Ranbow*** avoids searching the solutions space, instead using the multi-allelic variants to cluster the reads efficiently. For example, two bi-allelic polymorphic sites cannot cluster the reads into six classes in a hexaploid genome (2 × 2 possible sequence pattern). By integrating multi-allelic variants, one bi-allelic and one tri-allelic variant can provide six sequence patterns and can cluster the reads into six groups (2 × 3 possible sequence patterns). Multi-allelic variants enable ***Ranbow*** to cluster the reads and reconstruct local haplotypes. This approach is central to ***Ranbow***’s improved performance when compared to methods that search the solution space.
2. Integrating different types of errors: ***Ranbow*** integrates different types of errors simultaneously. It detects, corrects, or excludes erroneous reads. Excluding an erroneously mapped read, which is not a feature of methods using the MEC model, increases the speed and accuracy of our method. Methods using the MEC model have to integrate the erroneously mapped reads to one of the assembled haplotypes. Only ***Ranbow*** excludes these reads.
3. Data structure: ***Ranbow*** consists of local haplotype reconstruction steps followed by global haplotype elongation. In contrast to read-clustering and read-graph based methods, ***Ranbow*** compares only a local selection of reads in its first step. ***Ranbow***’s data structure facilitates random access to local regions with higher signal for partitioning reads. This is a distinct advantage over sliding window approaches and directly enables ***Ranbow*** to escape from local optimum solutions. In addition, ***Ranbow*** constructs a multipartite graph where the nodes are haplotype segments reconstructed from read clusters. The number of haplotype segments is limited by the ploidy of the organism. In contrast, read clustering or read-graph based methods use reads as the graph nodes. Increasing the sequencing depth is computationally expensive and negatively affects the performance of these methods. ***Ranbow*** uses the reads as an entity stemming from one haplotype. Variant-based graph methods shear the reads into variant connections. One read covering three variants becomes three bi-variant connections in these approaches. These methods rely on computational steps to connect these variants again.

### Conclusion

We have addressed the challenge of haplotype reconstruction for polyploid organisms from short read data. We investigated the characteristics of polyploid genomes and the technical challenges for reconstructing haplotypes. We introduced the AoM problem, and discussed how this, combined with the higher search space and the high error rate, are the main technical challenges when reconstructing haplotypes from sequence data. We designed ***Ranbow***, a method that embeds multiple error correction steps to detect different types of errors, i.e. base-calling, variant-calling, and mapping errors. Our method successfully integrates bi-allelic and multi-allelic variants. Although multi-allelic variants inflate the search space, the ***Ranbow*** algorithm is independent of this parameter, enabling it to reconstruct haplotypes quickly. ***Ranbow*** does not use dosage information as input, yet can be used for calling dosage information when haplotypes are computed. This is advantageous because in addition to read frequency, the information on which flanking variants are connected to each other through sequence reads is also taken into account.

Our work shows that ***Ranbow*** generally outperforms other haplotypers in terms of reconstructed haplotype length, accuracy, and computational and memory efficiency on real and simulated datasets. Only in a few datasets, the increase in accuracy and efficiency came at the cost of shorter haplotypes and increased gap sizes. ***Ranbow***’s general superior performance stems from the specific features and data structure we considered during the design process. It considers the AoM feature in every step and deal with different types of errors simultaneously. The erroneous reads are corrected or excluded within two layers of data structure, namely a local haplotype reconstruction followed by a global procedure. This data structure allows ***Ranbow*** to search for regions with better signals and prioritize them for the local haplotype reconstruction step. The local haplotype reconstruction step constructs haplotype segments in haplotype blocks. The global procedure combines these haplotype by building a multipartite graph.

We have shown that ***Ranbow*** is more than one order of magnitude faster than the competing approaches on real datasets, making whole genome haplotype reconstruction of complex genomes, such as the hexaploid sweet potato, possible. On a large computer cluster our method completed haplotype reconstruction of the sweet potato genome in approximately two days. This is a vast improvement on the estimated more than twenty days needed by the next best competing method. This feature allows the user to apply our method multiple times under different parameter settings or iteratively, e.g., for iterative assembly refinement [14]. We showed that our tool is able to efficiently utilize a combination of different insert sizes. This aligns with the established sequencing strategy of combining different insert lengths for *de novo* assembly of a new organism’s genome.

As long-read sequencing technologies become more wide-spread, there will be a need for programs that can assemble haplotypes efficiently and accurately. ***Ranbow*** fits the bill perfectly as it can also be applied to less heterozygous genomes. We hope to be able to improve ***Ranbow*** further by integrating base quality for long read sequencing, such as PacBio and Nanopore, and mapping quality and barcoded errors for linked reads, as well as the read count values. Our long term goal is that ***Ranbow*** will be the superior haplotyping method for a broad range of sequencing technologies.

## Supporting information

S1 Fig An illustration of *IHT* and *OHT* hash tables. This figure shows a mask (*mask* = (1, 4)), its seed sequence (‘11’,‘00’, and ‘10’) and the fragments containing these seed sequences (*f*_1_, *f*_2_, …, *f*_11_). For example, the *IHT* for the seed sequence ‘11’ contains its supporting fragments *IHT*(‘11’) = *f*_1_, *f*_2_, *f*_3_, *f*_4_. The *OHT* of a mask contains the *IHT*s of the mask seed sequences *OHT*(1, 4) = {*IHT*(‘11’),*IHT*(‘00’),*IHT*(‘10’)}.
(TIF)

S2 Fig Schematic view of graph *G*. This figure illustrates haplotype blocks and segments, and shows fragments and their corresponding edges in the graph. *G* is *k*-partite graph with haplotype blocks defining the partitions and the haplotype segments defining the nodes within the partitions. If two reads of a fragment are mapped to two haplotype segments of different blocks, an edge is assigned between the nodes of these segments.
(TIF)

S3 Fig Base quality and length distribution of Roche 454 reads. **Left**: Each 454 read is divided into 20 equal size segments. The box plot shows the base quality distribution and the red line indicates the high threshold we set for filtering the bases on quality. **Right**: The Roche 454 length distribution. Maximum length is 1771bp.
(TIF)

S4 Fig Selected scaffolds’ properties. In this heat map, each dot depicts one scaffold of the sweet potato genome. These scaffolds were obtained after the scaffolding step of *de novo* assembly. The x-axis shows the log scale of coverage and the y-axis shows the log scale of scaffold lengths. We randomly selected 50 scaffolds (red circles) of different sizes, namely 10kb, 50kb, 100kb, 500kb, and 1000kb, ten scaffolds each. These scaffolds are used for evaluations with real data and producing the simulated dataset.
(TIF)

S5 Fig Dataset properties of sweet potato genome. **Left**: Insert size distribution. The real data contains five different libraries with different insert sizes, namely 350bp, 550bp, 950bp, 20k, and no size selection. For simulated data, we generated inserts of 350bp from the selected scaffolds with 30x coverage for each haplotype. **Right**: Coverage of selected scaffolds for real and simulated data. The x-axis shows base coverage, and the y-axis depicts frequency. In the real dataset, the base coverage varies in a wide range up to 10k while the simulated data has the peak at 180x. This discrepancy is caused by the presence of repeats in the genome.
(TIF)

S6 Fig Insertion length distribution for simulated dataset of CBU genome. Four 100bp paired-end read libraries with insert sizes of 350bp, 1kbp, 2kbp and 5kb are generated by EAGLE (Enhanced Artificial Genome Engine) [45]. EAGLE generates reads and converts them to alignments. It is designed to simulate the behavior of Illumina’s Next Generation Sequencing instruments. For each library, the coverage for every haplotype is 40x.
(TIF)

S7 Fig Distribution of the number of variants covered by Roche 454 reads. (TIF)

S8 Fig Comparing the accuracy of the methods on real and simulated sweet potato data. The data are categorized into the five different categories: very short, short, medium-size, long, and very long. The real data does not contain the very long category because it is done by Roche 454 reads, which are limited in size.
(TIF)

S9 Fig *Ranbow* and *H-PoP* compared in terms of reconstructed haplotype length measured in base pairs. Data is based on *All*_350*bp*_ generated for CBU genome. Five violin plots represent measurements for five genome lengths and y-axis reports haplotype length in base pairs.
(TIF)

S10 Fig Length and frequency distribution of assembled haplotypes by different insert size on simulated sweet potato data. The x-axis shows different insert sizes. The y-axis depicts the number of matches in the assembled haplotype. The width of the violin plots shows the distribution of the number of assembled haplotypes within the groups. Except for the *350bp* group, in which ***H-PoP*** performs better, ***Ranbow*** outperforms in all groups including the collection of all insert sizes, which is depicted in the *All* group.
(TIF)

S11 Fig Match-mismatch plot for assembled haplotypes using Roche 454 reads. The color of each dot represents how many pairs of 454 reads with its corresponding assembled haplotype have a certain number of matches (*x*-axis) and mismatches (*y*-axis).
(TIF)

S1 Text Simulated data. This section explains the procedure for generating simulated data.
(PDF)

S2 Text Usability of different tools. This section compares the usability of competing methods.
(PDF)

S1 TableRunning time in second for $All_{R}^{b}$ and *All*_350*bp*_ datasets.(PDF)
