# Supplementary figures and images for: Ranbow: A fast and accurate method for polyploid haplotype reconstruction
Source: PLoS Comput Biol. 2020 May 29;16(5):e1007843. doi: 10.1371/journal.pcbi.1007843 (PMC7310859; doi:10.1371/journal.pcbi.1007843)

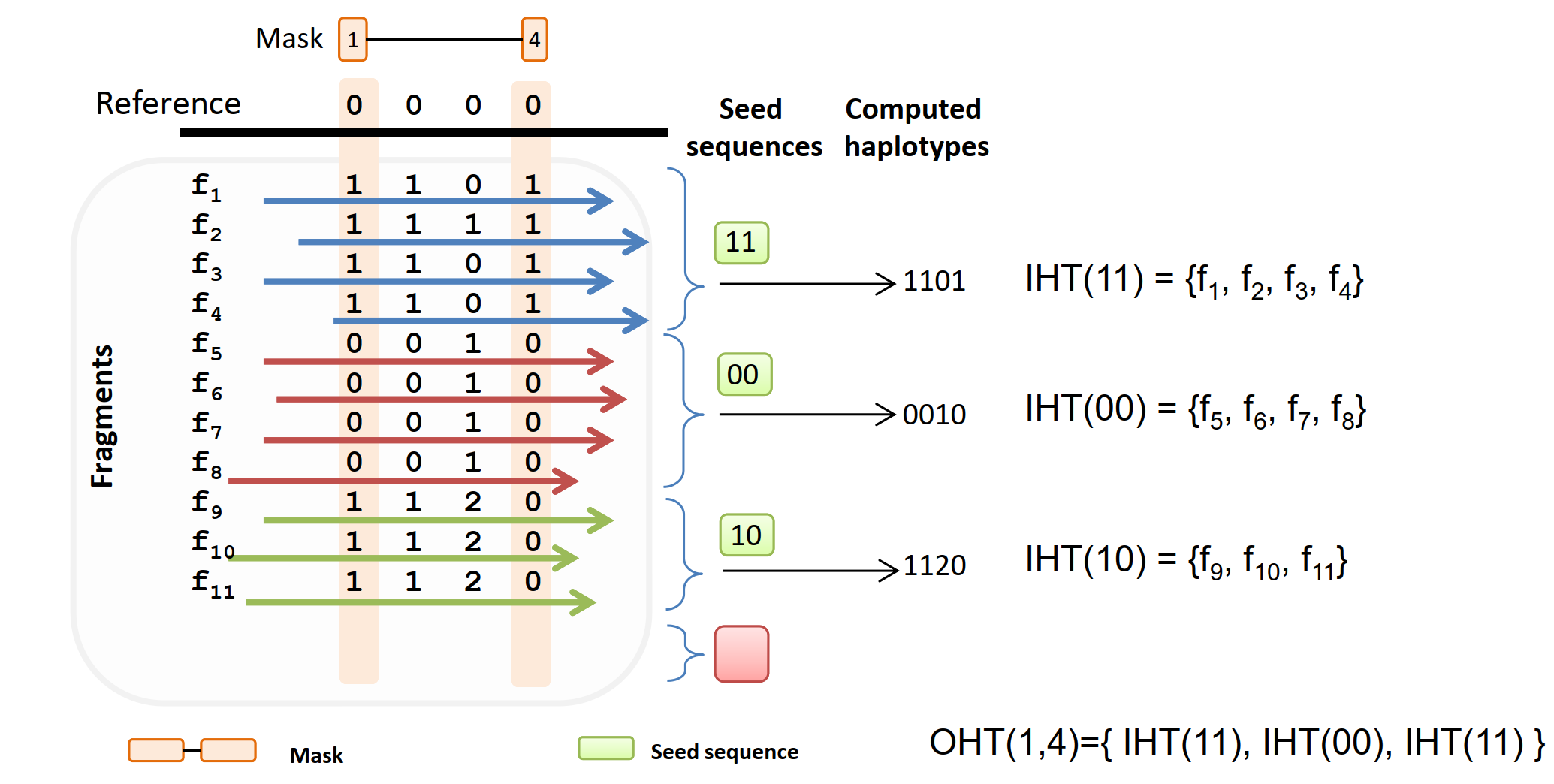

Supplement: S1 Fig — This figure shows a mask (mask = (1, 4)), its seed sequence (‘11’,‘00’, and ‘10’) and the fragments containing these seed sequences (f1, f2, …, f11). For example, the IHT for the seed sequence ‘11’ contains its supporting fragments IHT(‘11’) = f1, f2, f3, f4. The OHT of a mask contains the IHTs of the mask seed sequences OHT(1, 4) = {IHT(‘11’),IHT(‘00’),IHT(‘10’)}. (TIF) [file pcbi.1007843.s001.tif]

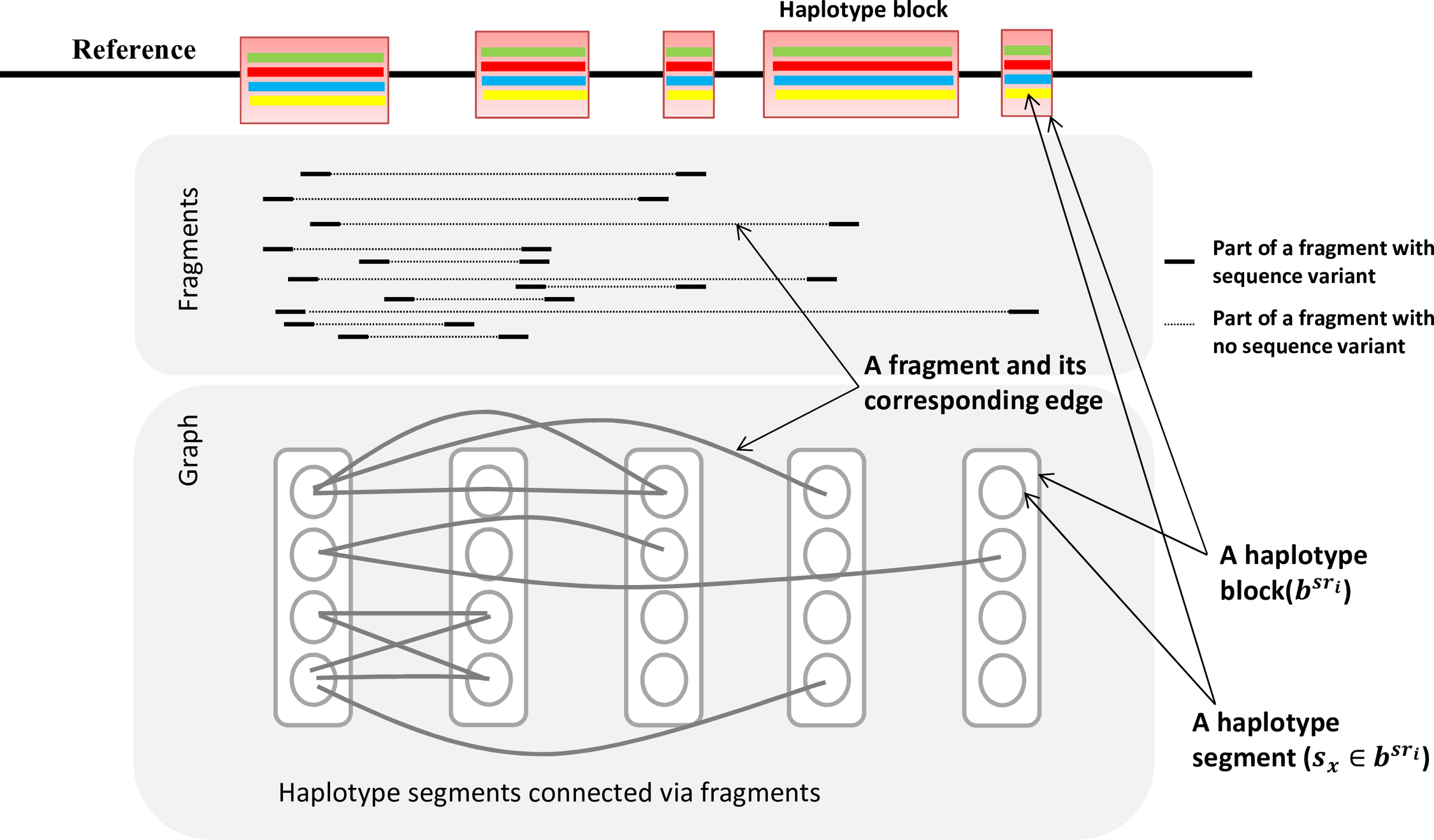

Supplement: S2 Fig — This figure illustrates haplotype blocks and segments, and shows fragments and their corresponding edges in the graph. G is k-partite graph with haplotype blocks defining the partitions and the haplotype segments defining the nodes within the partitions. If two reads of a fragment are mapped to two haplotype segments of different blocks, an edge is assigned between the nodes of these segments. (TIF) [file pcbi.1007843.s002.tif]

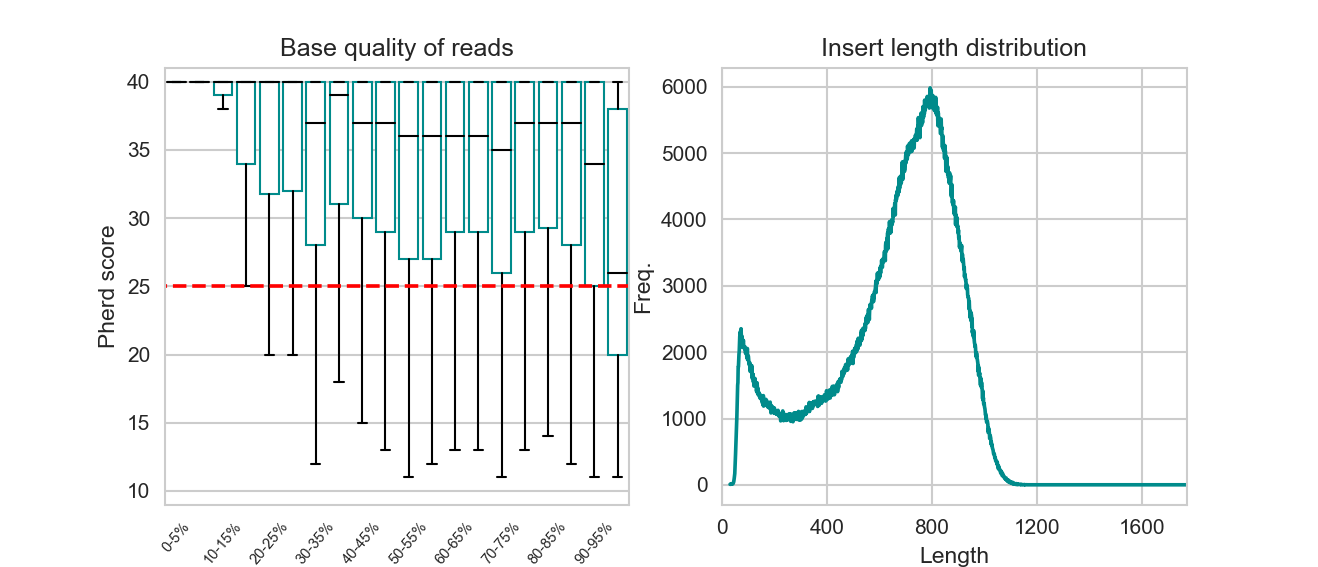

Supplement: S3 Fig — Left: Each 454 read is divided into 20 equal size segments. The box plot shows the base quality distribution and the red line indicates the high threshold we set for filtering the bases on quality. Right: The Roche 454 length distribution. Maximum length is 1771bp. (TIF) [file pcbi.1007843.s003.tif]

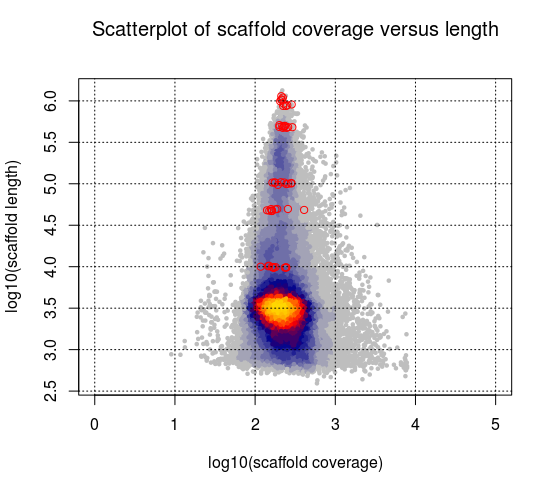

Supplement: S4 Fig — In this heat map, each dot depicts one scaffold of the sweet potato genome. These scaffolds were obtained after the scaffolding step of de novo assembly. The x-axis shows the log scale of coverage and the y-axis shows the log scale of scaffold lengths. We randomly selected 50 scaffolds (red circles) of different sizes, namely 10kb, 50kb, 100kb, 500kb, and 1000kb, ten scaffolds each. These scaffolds are used for evaluations with real data and producing the simulated dataset. (TIF) [file pcbi.1007843.s004.tif]

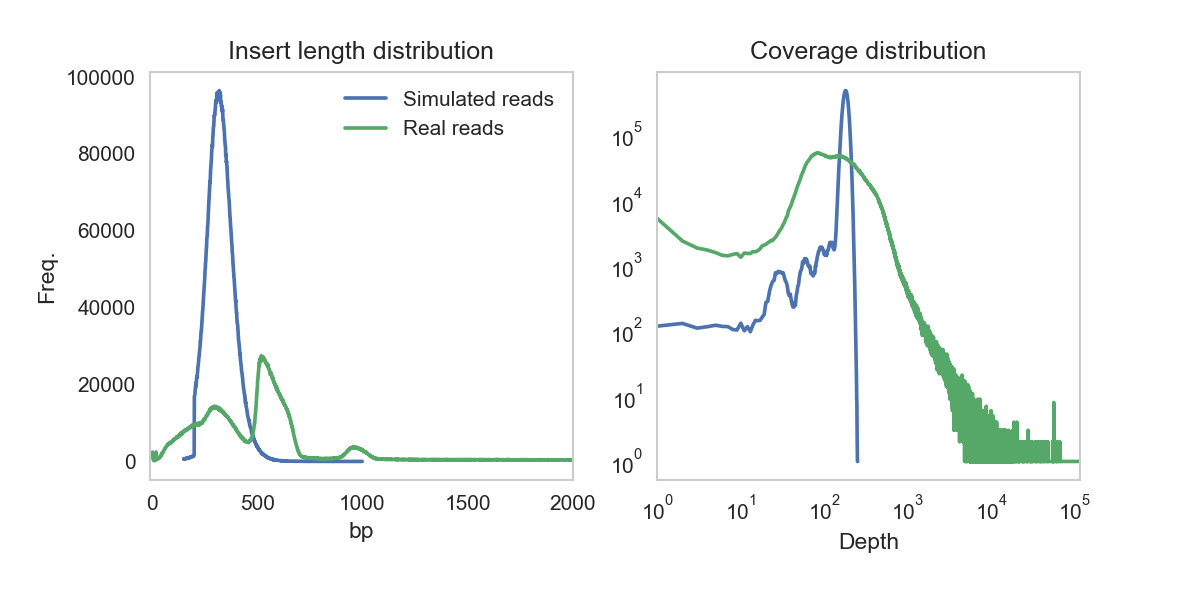

Supplement: S5 Fig — Left: Insert size distribution. The real data contains five different libraries with different insert sizes, namely 350bp, 550bp, 950bp, 20k, and no size selection. For simulated data, we generated inserts of 350bp from the selected scaffolds with 30x coverage for each haplotype. Right: Coverage of selected scaffolds for real and simulated data. The x-axis shows base coverage, and the y-axis depicts frequency. In the real dataset, the base coverage varies in a wide range up to 10k while the simulated data has the peak at 180x. This discrepancy is caused by the presence of repeats in the genome. (TIF) [file pcbi.1007843.s005.tif]

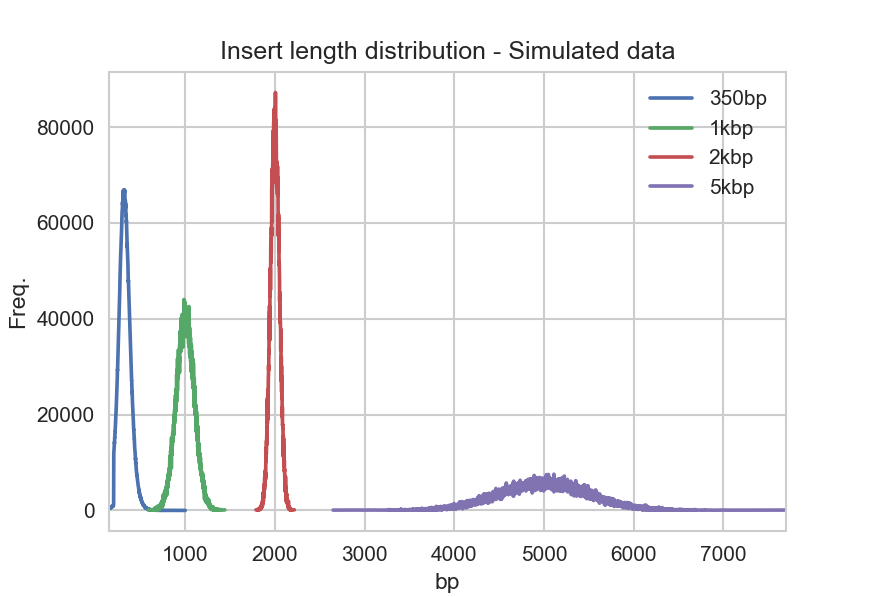

Supplement: S6 Fig — Four 100bp paired-end read libraries with insert sizes of 350bp, 1kbp, 2kbp and 5kb are generated by EAGLE (Enhanced Artificial Genome Engine) [45]. EAGLE generates reads and converts them to alignments. It is designed to simulate the behavior of Illumina’s Next Generation Sequencing instruments. For each library, the coverage for every haplotype is 40x. (TIF) [file pcbi.1007843.s006.tif]

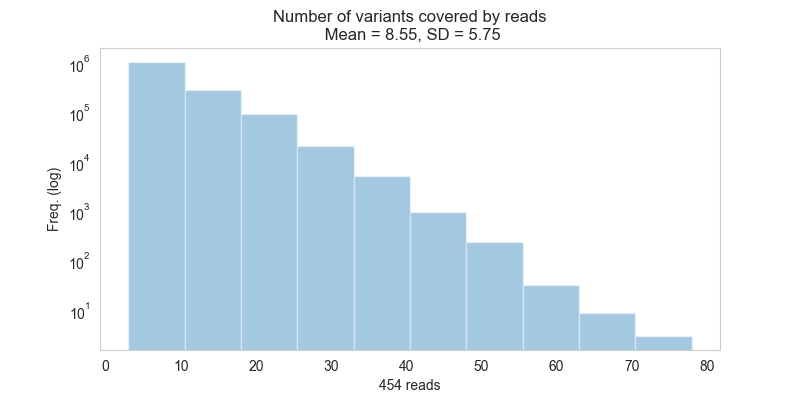

Supplement: S7 Fig — (TIF) [file pcbi.1007843.s007.tif]

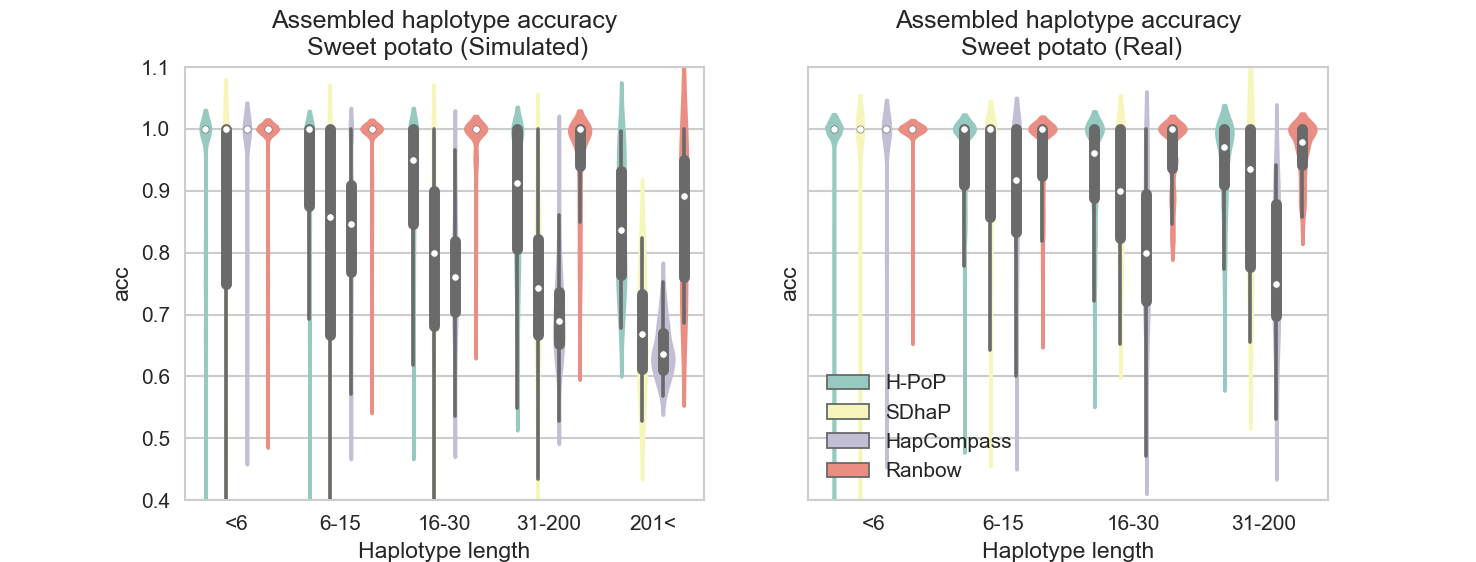

Supplement: S8 Fig — The data are categorized into the five different categories: very short, short, medium-size, long, and very long. The real data does not contain the very long category because it is done by Roche 454 reads, which are limited in size. (TIF) [file pcbi.1007843.s008.tif]

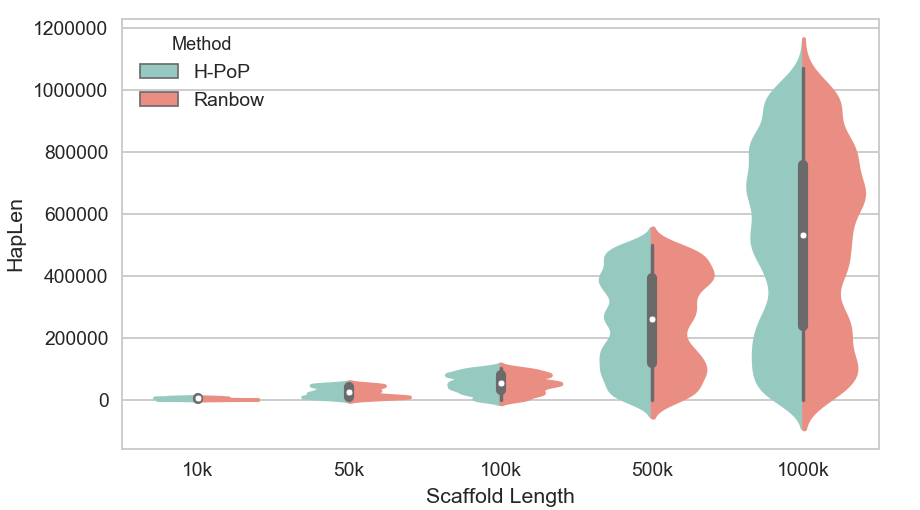

Supplement: S9 Fig — Data is based on All350bp generated for CBU genome. Five violin plots represent measurements for five genome lengths and y-axis reports haplotype length in base pairs. (TIF) [file pcbi.1007843.s009.tif]

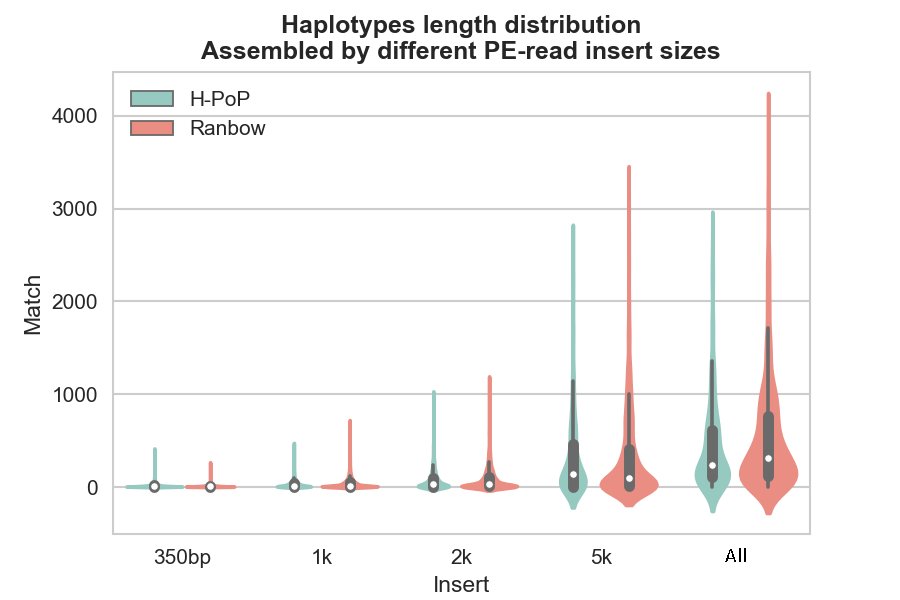

Supplement: S10 Fig — The x-axis shows different insert sizes. The y-axis depicts the number of matches in the assembled haplotype. The width of the violin plots shows the distribution of the number of assembled haplotypes within the groups. Except for the 350bp group, in which H-PoP performs better, Ranbow outperforms in all groups including the collection of all insert sizes, which is depicted in the All group. (TIF) [file pcbi.1007843.s010.tif]

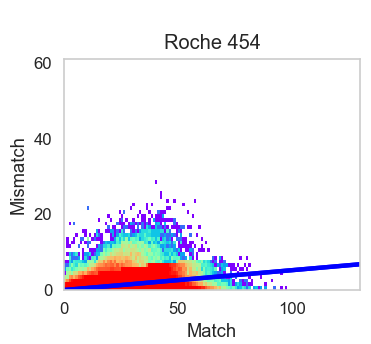

Supplement: S11 Fig — The color of each dot represents how many pairs of 454 reads with its corresponding assembled haplotype have a certain number of matches (x-axis) and mismatches (y-axis). (TIF) [file pcbi.1007843.s011.tif]
