# Supplementary material for: Ranbow: A fast and accurate method for polyploid haplotype reconstruction
Source: PLoS Comput Biol. 2020 May 29;16(5):e1007843. doi: 10.1371/journal.pcbi.1007843 (PMC7310859; doi:10.1371/journal.pcbi.1007843)
Supplement: S1 Text — This section explains the procedure for generating simulated data. (PDF) [file pcbi.1007843.s012.pdf]

Supporting information

Simulated data

Several goals can be achieved through the use of simulated data:

- To measure the haplotype assembly accuracy using known haplotypes of an organism
- To eliminate the influence of the reads alignment process and variant calling.
- To preserve all characteristics of an organism's genome, such as:
  - the genomic region length distribution,
  - the genotype distribution, and
  - the intervals between polymorphic sites.

To achieve these goals, six haplotypes were generated based on real scaffolds and VCF files of *Ipomoea batatas*. For example, the position 100 is a start of a polymorphic site; the reference allele is $ATGA$, the alternative alleles are $ATGTA$, $ATGTTA$, $ATGTTTA$ with genotype 0/0/0/1/2/3. Three haplotypes have reference allele $ATGA$ starting with position 100. One haplotype has the first alternative allele $ATGTA$, one haplotype has the second alternative allele $ATGTTA$, and one haplotype the third alternative allele $ATGTTTA$. The six generated haplotypes were saved in a FASTA format.

The sequence read simulator EAGLE (Enhanced Artificial Genome Engine) was used to generate reads and convert them to alignments. EAGLE is designed to simulate the behavior of Illumina's Next Generation Sequencing instruments. It can simulate random base-calls with properties similar to real datasets. EAGLE was run with six haplotypes to generate paired-end reads with 100bp length. The coverage of every haplotype was set to 30, resulting in a mean dataset coverage depth of 180. An alignment file is generated without aligning the reads because the simulated read origin is known. This enabled us to preserve the original characteristics of the organism's sequence, meaning that true haplotypes could be compared for the purposes of evaluation.

The same data preprocessing steps as for real data were done for simulated data. The evaluation algorithm was changed because true haplotypes were available. The assembled haplotypes were mapped to the true haplotypes, instead of trying to map the sequence reads to assembled haplotypes. The output data of the evaluation algorithm had the same structure as the real data evaluation output.

Algorithm 1: Haplotypes evaluation algorithm

Input: reconstructed haplotypes set H, evaluation alignments set E, polymorphic sites set P, $P = \{p_0, p_1, ..., p_{m-1}\}$, $p = (position, (a_0, a_1, ..., a_{k-1}))$, where $m = |P|$, a_0 - reference allele, $a_1, ..., a_{k-1}$ - alternative alleles, k - ploidy number

Output: $n2$ table with $n = |H|$, 1st column $= \#Match$, 2nd column $= \#Mismatch$

```
begin
    foreach alignment e ∈ E do
        if e covers at least two polymorphic sites then
            H' = [ ];
            foreach haplotype h ∈ H do
                if e and h have at least two common polymorphic sites then
                    H' ← h
            match, mismatch = scoring_function(e, H', P);
            write match and mismatch to the output table;
end
```

Algorithm 2: Scoring function

```
Input: reconstructed haplotypes set H', evaluation alignment e,
       polymorphic sites set P
Output: #Match, #Mismatch
begin
    best_score = −∞;
    best_score_match = 0;
    best_score_mismatch = 0;
    foreach h ∈ H' do
        match = 0;
        mismatch = 0;
        P_common = {p | p ∈ P ∧ p_position ∈ h_positions ∧ p_position ∈
        e_positions};
        foreach p ∈ P_common do
            if allele^e_position ∈ p_alleles ⋀ allele^h_position ∈ p_alleles then
                if allele^e_position = allele^h_position then
                    match = match + 1;
                else
                    mismatch = mismatch + 1;
        if match + mismatch ≥ 2 then
            score = match − mismatch^2;
            if score > best_score then
                best_score = score;
                best_score_match = match;
                best_score_mismatch = mismatch;
    return best_score_match, best_score_mismatch
end
```
