# Supplementary material for: Ranbow: A fast and accurate method for polyploid haplotype reconstruction
Source: PLoS Comput Biol. 2020 May 29;16(5):e1007843. doi: 10.1371/journal.pcbi.1007843 (PMC7310859; doi:10.1371/journal.pcbi.1007843)
Supplement: S2 Text — This section compares the usability of competing methods. (PDF) [file pcbi.1007843.s013.pdf]

Supporting information

Usability of different tools

The tools' usability was evaluated using the following parameters:

- Data preprocessing complexity: how much work should be done on the input data before the tool can process it;
- Data post-processing complexity: how much work should be done on the tool's output data to convert it to the desired format, e.g., to use it in the analysis;
- Complex polymorphism handling: whether the tool can process reads with insertions, deletions, multi-allelic nucleotide polymorphisms, and other complex polymorphic sites;
- Multi-allelic sites handling: the tool distinguishes between alternative alleles;

All the tools use a polymorphism-fragment matrix, yet the amount of work necessary to preprocess data differs significantly. Ranbow and HapCompass take a coordinate sorted alignment file in BAM format and VCF, internally converting them to a polymorphism-fragment matrix, whereas H-PoP and SDhaP leave the matrix preprocessing to the user. Due to the absence of any scripts or instructions for constructing such a matrix, this task may not be trivial for the inexperienced user. Ranbow users must be aware that the VCF must contain a CIGAR (Compact Idiosyncratic Gapped Alignment Report) string in the INFO column, the absence of which will lead to failure during the program's execution. While some variant callers add the CIGAR string by default, others do not. GATK does not provide the option of adding the CIGAR string in the INFO column. This condition therefore results in an additional VCF preprocessing step prior to using the current version of Ranbow.

The most useful data formats for storing haplotype information are FASTA, BAM, and VCF. Ranbow and HapCompass provide assembled haplotypes in FASTA and VCF formats, with Ranbow also generating a BAM file. H-PoP and SDhaP, in contrast, produce custom format files, containing start and end positions of assembled haplotype blocks, the start and end allele in every block, and a list of alleles in each block. Conversion into another format may be required depending on the user's next task.

Ranbow and HapCompass can process complex polymorphic sites and internally convert them to the required format, whereas H-PoP and SDhaP do not accept complex polymorphic sites by default. In the case of H-PoP, it is possible to manually convert the complex polymorphisms and corresponding

base qualities to a single character, representing either reference or alternative allele, and single base quality by taking the average over all bases forming the polymorphism. SDhaP offers no trivial solutions for complex polymorphisms because it requires the exact allele base, as opposed to reference or alternative allele information, as an input.

For diploid organisms it is sufficient to know whether the haplotype has a reference or an alternative allele on a site. Because polyploid organisms may have different alternative alleles, it is essential to know which alternative allele has a haplotype. With respect to multi-allelic sites handling, Ranbow is superior because the output haplotypes contain exact information about alternative alleles. SDhaP shows the exact alternative allele because the alleles in its input and output matrix are encoded with nucleotides, meaning that SDhaP cannot distinguish between reference and alternative alleles. As SDhaP can not process complex polymorphic sites, the advantage is lost for organisms with a ploidy number higher than 4. H-PoP and HapCompass do not specify the alternative allele in their output, so the accuracy of the assembled haplotypes may be decreased if the organism's genome is highly heterozygous and contains a high number of multi-allelic polymorphic sites.
