# Supplementary material for: Ranbow: A fast and accurate method for polyploid haplotype reconstruction
Source: PLoS Comput Biol. 2020 May 29;16(5):e1007843. doi: 10.1371/journal.pcbi.1007843 (PMC7310859; doi:10.1371/journal.pcbi.1007843)
Supplement: S1 Table — (PDF) [file pcbi.1007843.s014.pdf]

# Supporting information

## Supplementary table

**S1 Table** Running time in second for $All_R^b$ and $All_{350bp}$ datasets.

| | **Real**($All_R^b$) | | | | | **Simulated**($All_{350bp}$) | | | | |
|---|---|---|---|---|---|---|---|---|---|---|
| | 10kbp | 50kbp | 100kbp | 500kbp | 1Mb | 10kbp | 50kbp | 100kbp | 500kbp | 1Mb |
| **Ranbow** | 0.3 | 10.3 | 46.4 | 776.3 | 3071.2 | 0.8 | 4.4 | 11.3 | 46.6 | 98.6 |
| **SDhaP** | 432.5 | 10156.8 | 12035.2 | 13680.5 | - | 154.9 | 480.7 | 1330.4 | 5010.0 | 7150.2 |
| **H-PoP** | 10.5 | 89.3 | 278.3 | 3752.3 | 22586.8 | 10.1 | 39.5 | 96.8 | 338.3 | 664.4 |
| **HapCompass** | 350.7 | 3033.5 | 56921.1 | - | - | 11.1 | 36.0 | 94.5 | 368.3 | 788.5 |
